# Lipid environment modulates processivity and kinetics of a presenilin homolog acting on multiple substrates *in vitro*

**DOI:** 10.1016/j.jbc.2023.105401

**Published:** 2023-10-29

**Authors:** Yuqi Wu, Gwendell M. Thomas, Max Thomsen, Sara Bahri, Raquel L. Lieberman

**Affiliations:** School of Chemistry & Biochemistry, Georgia Institute of Technology, Atlanta, Georgia, USA

**Keywords:** intramembrane proteolysis, aspartic protease, proteomics mass spectrometry, enzyme kinetics, membrane biophysics

## Abstract

Intramembrane proteases (IPs) hydrolyze peptides in the lipid membrane. IPs participate in a number of cellular pathways including immune response and surveillance, and cholesterol biosynthesis, and they are exploited by viruses for replication. Despite their broad importance across biology, how activity is regulated in the cell to control protein maturation and release of specific bioactive peptides at the right place and right time remains largely unanswered, particularly for the intramembrane aspartyl protease (IAP) subtype. At a molecular biochemical level, different IAP homologs can cleave non-biological substrates, and there is no sequence recognition motif among the nearly 150 substrates identified for just one IAP, presenilin-1, the catalytic component of γ-secretase known for its involvement in the production of amyloid-β plaques associated with Alzheimer disease. Here we used gel-based assays combined with quantitative mass spectrometry and FRET-based kinetics assays to probe the cleavage profile of the presenilin homolog from the methanogen *Methanoculleus marisnigri JR1* as a function of the surrounding lipid-mimicking environment, either detergent micelles or bicelles. We selected four biological IAP substrates that have not undergone extensive cleavage profiling previously, namely, the viral core protein of Hepatitis C virus, the viral core protein of Classical Swine Fever virus, the transmembrane segment of Notch-1, and the tyrosine receptor kinase ErbB4. Our study demonstrates a proclivity toward cleavage of substrates at positions of low average hydrophobicity and a consistent role for the lipid environment in modulating kinetic properties.

Enzymatic proteolysis is a superficially simple process in which bulk water is activated for nucleophilic attack at the scissile bond of a substrate within the active site of a protease, and each protease active site contains signature features tailored to its substrate repertoire (1). Despite the requirement for activated water and apparent incompatibility of the hydrophobic lipid membrane, membrane-bound analogs of well-studied soluble proteases, so-called intramembrane proteases (IPs), are well known. Enzymatic proteolysis that occurs within the hydrophobic lipid nominally requires a complex among IP, transmembrane (TM) portion of a substrate, lipid bilayer, and bulk water. IPs underlie critical pathways including immune response and surveillance, cholesterol biosynthesis, and other major signaling pathways in the cell (2), and their associations with myriad diseases render them viable drug targets (3). The intramembrane aspartyl proteases (IAPs), which represent one of the four classes of IPs, use two membrane-embedded aspartates to catalyze the proteolysis of substrates, which are found in the conserved sequence motif YD..GXGD in which X is usually a hydrophobic residue such as Leu or Met (4). In contrast to soluble proteases (5), no consensus sequence recognition motif within the TM segment of IAP substrates has emerged (6). Thus, a major open question in the field is how proteolytic activity is regulated.

The primary members of the IAP subclass are signal peptide peptidase (SPP) and presenilin. SPP processes signal peptides after sheddase activity by the membrane-anchored signal peptidase (7), which releases remnant bioactive peptides from the membrane. The substrates for SPP include proteins involved in innate immunity, inflammation, and viral replication (8, 9, 10). Presenilin is the catalytic subunit of the γ-secretase complex and at last count, nearly 150 substrates have been identified (6). The best characterized presenilin substrates are amyloid precursor protein (APP) and Notch-1. Cleavage of the APP TM segment by γ-secretase releases different length amyloid-β (Aβ) peptides, including Aβ_40_ considered inert with respect to Alzheimer, Aβ_42_ found in senile plaques of Alzheimer patients and represents the γ-cleavage site, and Aβ_49_ representing ε-cleavage that is on-pathway to Aβ_42_ (11). Disease-causing mutations in presenilin or APP alter the levels and molecular properties of released Aβ peptides (12). Cleavage of Notch-1 by γ-secretase (13) is relatively less well-defined at a molecular level (14), but affects many fundamental processes associated with cell growth and differentiation, and dysregulation of cleavage leads to cancer and other diseases (15).

In this study, our goal was to evaluate how the biochemical properties of IAP cleavage are modulated by the lipidic environment (Fig. 1). We probed cleavage profiles and enzyme kinetics toward four biological substrates as a function of the lipid-mimicking environment, either detergent micelles or a characterized bicelle system, and employed the structurally characterized (16) presenilin homolog from the methanogen *Methanoculleus marisnigri JR1* (MCM.IAP, Uniprot A3CQV0, NCBI accession WP_011844759) (17). We selected four substrates total, two viral core proteins (10, 18) that are SPP substrates, as well as the TM segment of Notch-1 (13) and the tyrosine receptor kinase ErbB4 (19), which are presenilin substrates. To our knowledge, cleavage of viral core proteins has been examined only to a limited extent (10, 18, 20), and cleavage sites of ErbB4 have not been examined even though the majority of the 55 tyrosine receptor kinases are believed to be presenilin substrates (21). Our study demonstrates a preference of MCM.IAP toward cleavage of substrates at positions of low average hydrophobicity and a critical role for the lipidic milieu in modulating processivity and kinetics.

**Figure 1:**
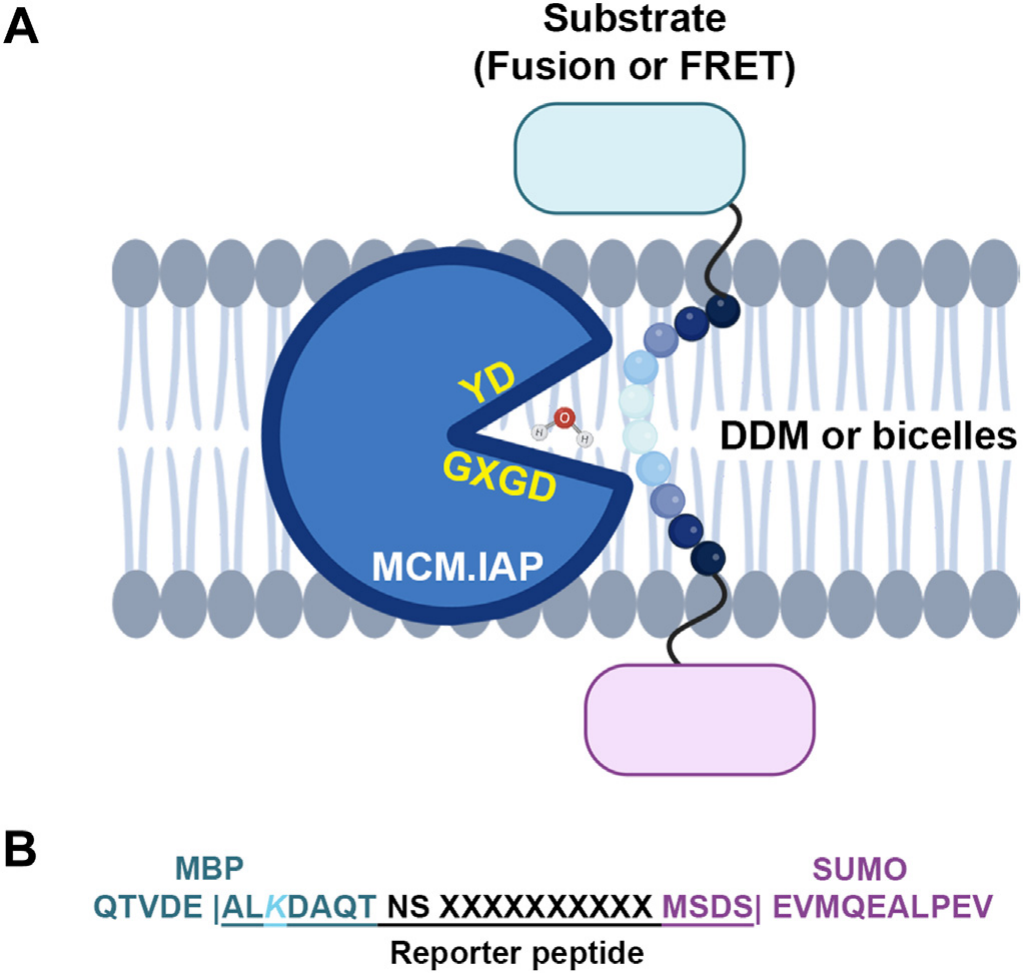
**Schematic representation of IAP reactivity and approach used in this study.***A*, IAPs use the aspartyl residues in the motif YD…GXGD and water to hydrolyze peptides within the lipid membrane. In this study, we probed the cleavage profiles and kinetics of the purified IAP family member MCM.IAP when reacted toward four selected substrate sequences, using either a fusion protein or FRET substrate. We also compared two lipid environment mimetics, DDM or bicelles. Our study shows modulation of the cleavage profile and kinetics by lipid as well as an overall cleavage preference at the site of hydrophobicity minimum (depicted as increasing lighter spheres toward the MCM.IAP active site) within the substrate. Figure created with BioRender.com license VW25TRO0NN. *B*, overview of MBP-ySUMO fusion peptide substrate. Reporter peptide bracketed with | and underlined. XXXXXXXXXX, 10 mer substrate sequence. *K*, lysine for ionization.

## Results

### Substrate selection and overall gel cleavage assay design

Initially, we scanned 148 γ-secretase substrates from the literature (6) to identify those with hydrophilic residues in register with the two major Thr-containing cut sites annotated for Aβ (Table 1). Sequence alignments were highly dependent on algorithms used and the presence or absence of sequences beyond regions of interest (not shown). Polar residues, when present, are generally not in register with each other. Only 20 of the 148 γ-secretase substrates from the literature could be aligned by any method such that hydrophilic residues were in register with the two major cut sites annotated for Aβ (Fig. S1).

**Table 1:** Substrates in this study

Context for the selected 10-residue peptide inserts (red) between fusion proteins MBP and ySUMO, predicted cleavage sites (underlined, *e.g.*, CLTF denotes cut sites C-L, L-T, and T-F), and observed cut sites in n-dodecyl-β-D-maltoside (DDM) and bicelles (one per line). See also Table S2.

We selected four representative substrates from the repertoire of reported substrates for human SPP or presenilin (Table 1), each with a polar (Cys, Ser, Thr) residue: the C-terminal transmembrane domain of the Hepatitis C Virus (HCV) core protein (10), the corresponding sequence from Classical Swine Fever Virus (CSFV) core protein (18), the C-terminal TM segment from receptor tyrosine kinase ErbB4, and the corresponding segment for Notch-1. For our established gel-based assay (22), a 10-residue sequence derived from the Uniprot-annotated TM helix of each substrate (23) was flanked with maltose binding protein (MBP) and yeast small ubiquitin-like modifier (ySUMO) (Fig. 1). Selected HCV and ErbB4 sequences have Ser or Thr, respectively, aligned with the Thr residue of previously examined Aβ (24), whereas CSFV and Notch-1 have Thr and Cys but do not align (Table S2).

Despite harboring only a short stretch of the TM segment, the fusion proteins extend from the substrate sequence in both directions, with two extra residues on the N-terminal side and 15 residues on the C-terminal side (Fig. 1*B*), which could not be modeled in the ySUMO crystal structure (25). The importance of the unstructured 15 residues at the start of ySUMO is underscored by the finding that replacement with human SUMO, which has a structured N-terminus, is inactive toward wild-type MCM.IAP (MCM.IAP^WT^, not shown). Small-angle X-ray scattering data of analogous substrates indicate an elongated bilobed solution structure (24). In sum, the MBP-ySUMO fusion construct setup is compatible with exposure of the 10-amino acid sequence of interest and accessibility to detergent, lipid, and MCM.IAP^WT^, without being aggregation-prone.

Each purified substrate was incubated with purified MCM.IAP^WT^ at a stoichiometry and time frame that maximizes the accumulation of cleavage products for identification of MCM.IAP cut site(s) by Glu-C digest followed by LC-MS/MS. Glu-C cleaves at Glu and Asp sites (26), with a strong preference for Glu (27). As such, our reporter peptide (Fig. 1*B*) is a peptide generated by cleavage at sequential glutamates [E].ALK…SDS.[E]. Robust ionization is endowed by the single unique Lys within this peptide (Fig. 1*B*). The potential alternative reporter peptide [D].AQT..S.[D] was observed only very rarely (average 0.5 PSM over 48 data sets obtained herein), and thus was not considered in the analysis. The abundance of each cleavage product was calculated as the ratio of the given reporter peptide cut site divided by total peptide spectral matches (PSMs) measured of any length within the reporter peptide, and the overall quality of the LC-MS/MS data was assessed by PSM values for five selected peptides from MBP (Table S1) (22). Reactions were performed with MCM.IAP purified in the presence of n-dodecyl-β-D-maltoside (DDM) micelles and/or reconstituted into bicelles composed of 1,2-dimyristoyl-*sn*-glycero-3-phosphocholine (DMPC) and 3-[(3-cholamidopropyl)dimethylammonio]-2-hydroxy-1-propanesulfonate (CHAPSO) (28) (Fig. 1*A*), previously used for MCM.IAP activity assays (22, 24). The morphology of the DMPC/CHAPSO bicelle changes depending on temperature and lipid composition (29), and adopts a cylindrical worm-like structure under the assay conditions used here (30).

### Cleavage patterns across four substrates assessed *via* gel-based assay

**Figure 2:**
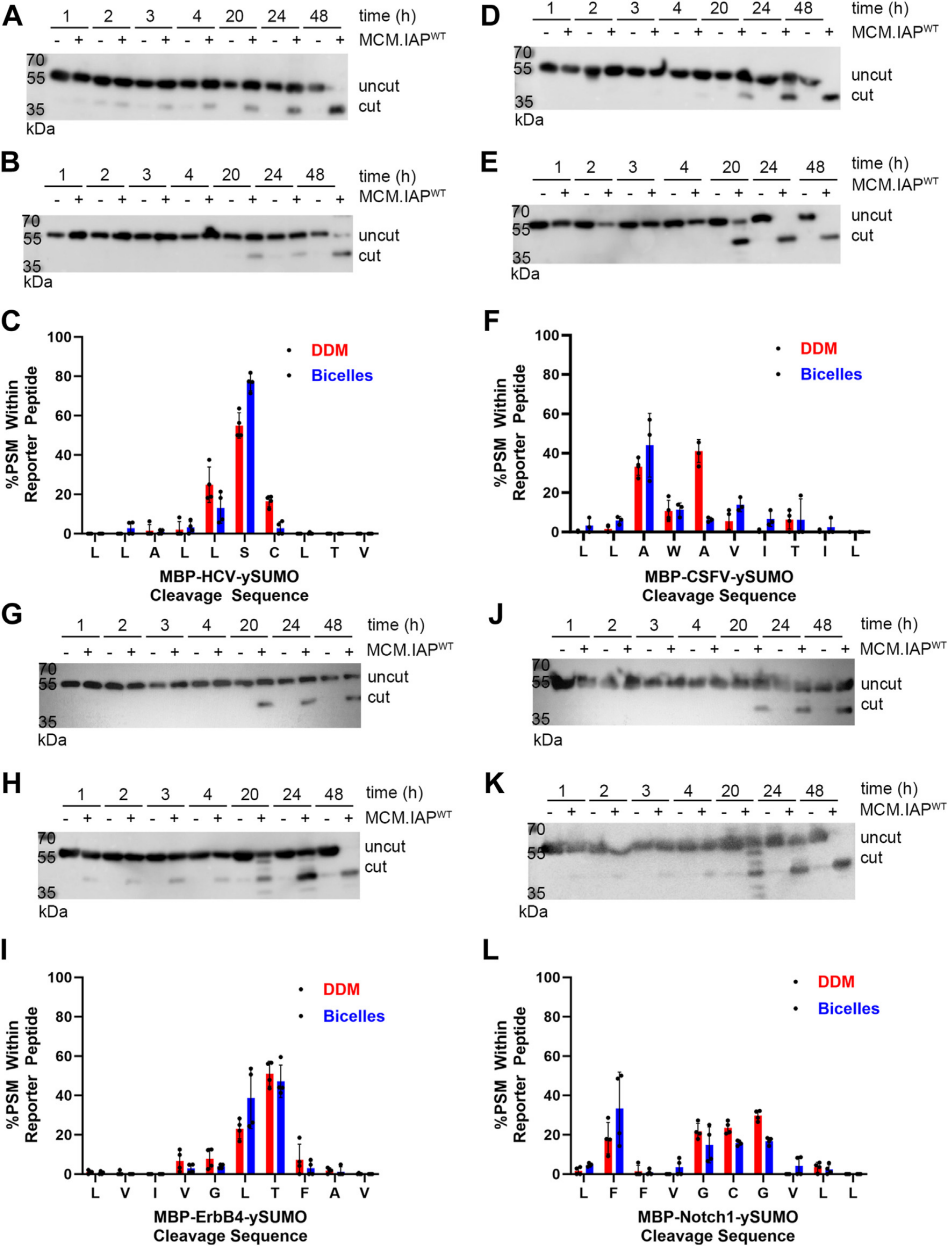
**MCM.IAPcleavageof MBP-HCV-ySUMO, MBP-CSFV-ySUMO, MBP-ErbB4-ySUMO and MBP-Notch-1-ySUMO.***A*, Western blot showing accumulation of cleavage product (∼40 kDa) of MBP-HCV-ySUMO over time in DDM and (*B*) in bicelles. *C*, LC-MS/MS cleavage profile for MBP-HCV-ySUMO. *D*, Western blot showing accumulation of cleavage product (∼40 kDa) of MBP-CSFV-ySUMO over time in DDM and (*E*) in bicelles. *F*, LC-MS/MS cleavage profile for MBP-CSFV-ySUMO. *G*, Western blot showing increased cleavage product of MBP-ErbB4-ySUMO over time in DDM and (*H*) in bicelles. *I*, LC-MS/MS cleavage profile for MBP-ErbB4-ySUMO. *J*, Western blot showing increased cleavage product of MBP-Notch-1-ySUMO over time in DDM and (*K*) in bicelles. *L*, the LC-MS/MS cleavage profile for MBP-Notch-1-ySUMO. Each LC-MS/MS profile contains at least two biological replicates. Error bars indicate standard deviation from all analytical replicates. See Table S1 for LC-MS/MS data and Fig. S2 for full Western blots. In these experiments, only the primary 40 kDa product is analyzed by LC-MS/MS, not the low-level spurious other cleavage products sometimes detected in Western blot.

MCM.IAP cleaved all four substrates and formed N-terminal cleavage products over time as visualized by an anti-MBP Western blot (Fig. 2 where panels *A*–*F* show results for SPP substrates and *G*–*L* show results for presenilin substrates); no degradation product is detected for any substrate in the same timeframe. MBP-HCV-ySUMO (Fig. 2, *A* and *B*) is the only substrate tested in this study where cleavage products appeared at the same qualitative rate in DDM or bicelles; accumulation of cleavage products for MBP-CSFV-ySUMO (Fig. 2, *D* and *E*), MBP-ErbB4-ySUMO (Fig. 2, *G*, *H*, *I* and *K*), and MBP-Notch-1-ySUMO (Fig. 2, *J* and *K*) occurred earlier when the reaction was conducted in bicelles compared to DDM. For SPP substrates (Fig. 2, *A*–*F*), MBP-HCV-ySUMO and MBP-CSFV-ySUMO, cleavage patterns are dissimilar, but cleavage appeared more precise in bicelles for both substrates. Namely, in DDM, the main cleavage sites for MBP-HCV-ySUMO were L5-S6 and S6-C7 whereas S6-C7 was clearly dominant in bicelles (Fig. 2*C*). Similarly, for MBP-CSFV-ySUMO, the cut sites in DDM are predominantly at A3-W4 and A5-V6 (Fig. 2*F*) whereas in bicelles, A3-W4 dominated and A5-V6 was minor in comparison (Fig. 2*F*). For presenilin substrates (Fig. 2, *G*–*L*), MBP-ErbB4-ySUMO and MBP-Notch-1-ySUMO, cleavage patterns were again dissimilar, but the effect of bicelles was less pronounced than for SPP substrates. ErbB4 had major cut sites at L6-T7 and T7-F8, in both DDM and bicelle conditions (Fig. 2*I*). For Notch-1 there was no dominant cut site: F2-F3, G5-C6, C6-G7, and G7-V8 were all observed to a similar extent regardless of the amphiphilic milieu (Fig. 2*L*). In sum, two substrates, ErbB4 and HCV, which contain Ser or Thr at position 6 or 7, cleave relatively specifically, as our Aβ substrates (Table 1 and Table S2) (22, 24), whereas the presence of Cys at position 6 or 7, does not have a similar effect.

### Role of Phe and Cys within Notch-1 substrate in guiding MCM.IAP cleavage products

**Figure 3:**
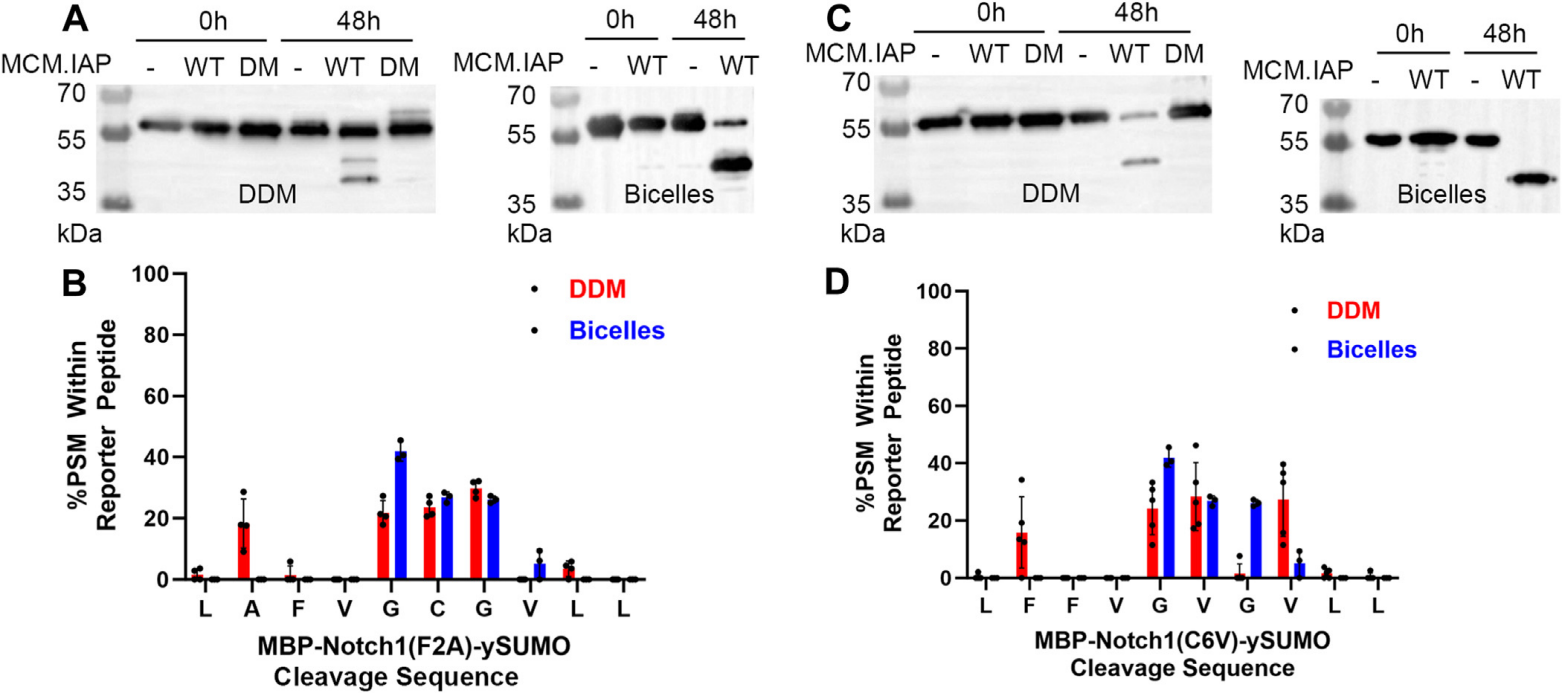
**MCM.IAP cleavage ofMBP-Notch-1(F2A)-ySUMO and MBP-Notch-1(C6V)-ySUMO mutants.***A*, anti-MBP Western blot shows accumulation of N-terminal cleavage products of MBP-Notch-1(F2A)-ySUMO by MCM.IAP^WT^ after incubation for 48 h at 37 °C in DDM (*left*) and bicelles (*right*). No product was observed for negative control or MCM.IAP double mutant (MCM.IAP^DM^). *B*, LC-MS/MS cleavage profile for MBP-Notch-1(F2A)-ySUMO in DDM and bicelles. *C*, anti-MBP Western blot shows the accumulation of N-terminal cleavage products of MBP-Notch-1(C6V)-ySUMO by MCM.IAP after incubation for 48 h at 37 °C, and no product for negative control or MCM.IAP^DM^ in DDM (*left*) and bicelles (*right*). *D*, LC-MS/MS cleavage profile for MBP-Notch-1(C6V)-ySUMO cleavage products. Each LC-MS/MS profile contains at least two biological replicates. Error bars indicate standard deviation from all analytical replicates. See Table S1 for LC-MS/MS data and Fig. S2 for full Western blots. In these experiments, only the primary 40 kDa product is analyzed by LC-MS/MS, not the low-level spurious other cleavage products sometimes detected in Western blot.

To further probe the apparent promiscuity of MCM.IAP toward MBP-Notch-1-ySUMO, we focused on cleavage at F2-F3 and G5-C6-G7. First, we mutated the scissile S1 site Phe to Ala (F2A) to probe the effect of the bulky hydrophobic residue at this position. MCM.IAP cleaved this substrate, and the products accumulated qualitatively faster in bicelles as for the parent substrate (Fig. 3*A*), but cleavage was not observed at position A2-F3 (Fig. 3*B*), indicating a chemical preference for Phe at this position. Next, we mutated the Cys to Val (C6V), a residue more prevalent among TM helices compared to Cys (31). Again, cleavage products were detected, and the accumulation was faster in bicelles (Fig. 3*C*). Cleavage at V6-G7 was similar to C6-G7, but cleavage at the original G7-V8 site was abrogated (Fig. 3*D*). In DDM, accumulation of a cut product at V8-L9 was observed. In bicelles, some new cleavage was observed at L1-F2 and at L9-L10, albeit at low abundance. Reactions were only seen in the presence of MCM.IAP^WT^; no cleavage was seen for the catalytic double mutant (D162A/D220A, DM), MCM.IAP^DM^. The results support the importance of Cys in Notch-1 in directing positional processing by MCM.IAP but in a manner different from the role of Thr in other substrates tested here and previously (22, 24).

### Comparison of MCM.IAP kinetics toward ErbFRET and NotchFRET in DDM *versus* bicelles

Given the distinct cleavage profiles for MBP-HCV-ySUMO and MBP-ErbB4-ySUMO *versus* MBP-CSFV-ySUMO and MBP-Notch-1-ySUMO in the gel assay, we next sought to compare enzyme kinetics, using the FRET-based assay described previously (22, 24) (Fig. 1). FRET substrates for the ErbB4 and Notch-1 were successfully synthesized (not shown). ErbFRET contains the 10-mer insert from MBP-ErbB4-ySUMO, and NotchFRET contains the 10-mer insert from MBP-Notch-1-ySUMO (Table 2). FRET assays were set up under pseudo-first order conditions for Michaelis-Menten analysis with a minimum 2× excess substrate over enzyme and analyzed as described previously (22, 24).

**Table 2:** Kinetic parameters of C100FRET, ErbFRET, and NotchFRET cleaved by MCM.IAP^WT^ in DDM and bicelles and inhibition by noted fold-excess inhibitor, ZLL_2_ketone

Substrate	(ZLL)_2_ ketone	K_m_ (μM)	V_max_ (nM min^−1^)	k_cat_ × 10^−3^ (min^−1^)	(k_cat_/K_m_) × 10^−3^ (μM^−1^•min^−1^)
DDM					
C100FRET NMA-GGVVIATV-DNP^a^	-	7.5 ± 1.0	11.2 ± 0.6	22.3 ± 1.2	3.0 ± 0.10
ErbFRET EDANS-LVIVGLTFAV-DABCYL^b^	-	4.9 ± 0.6	1.4 ± 0.05	2.8 ± 0.10	0.57 ± 0.07
	2×	6.0 ± 1.2	1.2 ± 0.10	2.4 ± 0.20	
	5×	7.7 ± 1.5	1.4 ± 0.10	2.8 ± 0.20	
	10×	11 ± 2	1.6 ± 0.10	3.1 ± 0.30	
NotchFRET EDANS-FFVGCGVLL-DABCYL	-	4.2 ± 0.5	0.98 ± 0.04	2.0 ± 0.10	0.48 ± 0.06
	2×	5.9 ± 0.6	0.8 ± 0.03	1.5 ± 0.10	
	5×	6.8 ± 0.9	0.8 ± 0.04	1.6 ± 0.10	
	10×	9.3 ± 1.2	0.8 ± 0.05	1.6 ± 0.10	
ReninFRET EDANS-IHPFHLVIHT - DABCYL (data from ref (23))		5.2 ± 0.5	2.25 ± 0.07	4.6 ± 0.30	0.88 ± 0.13
Bicelles					
C100FRET		2.8 ± 0.3	11.3 ± 0.30	22.7 ± 0.70	8.1 ± 0.10
ErbFRET		6.3 ± 0.6	4.6 ± 0.20	9.2 ± 0.30	1.5 ± 0.20
NotchFRET		8.3 ± 0.8	6.9 ± 0.30	13.8 ± 0.50	1.7 ± 0.20
ReninFRET (data from ref (23))		5.1 ± 0.6	2.16 ± 0.09	4.3 ± 0.40	0.84 ± 0.10

a NMA, N-methyl anthranilate (donor), DNP, lysine-modified with 2-nitrophenol (quencher).

b EDANS, N-[2-[(5-sulfo-1-naphthalenyl)amino]ethyl]-L-α-glutamine (donor), DABCYL, 4-((4-(dimethylamino)phenyl)azo)benzoyl (quencher).

**Figure 4:**
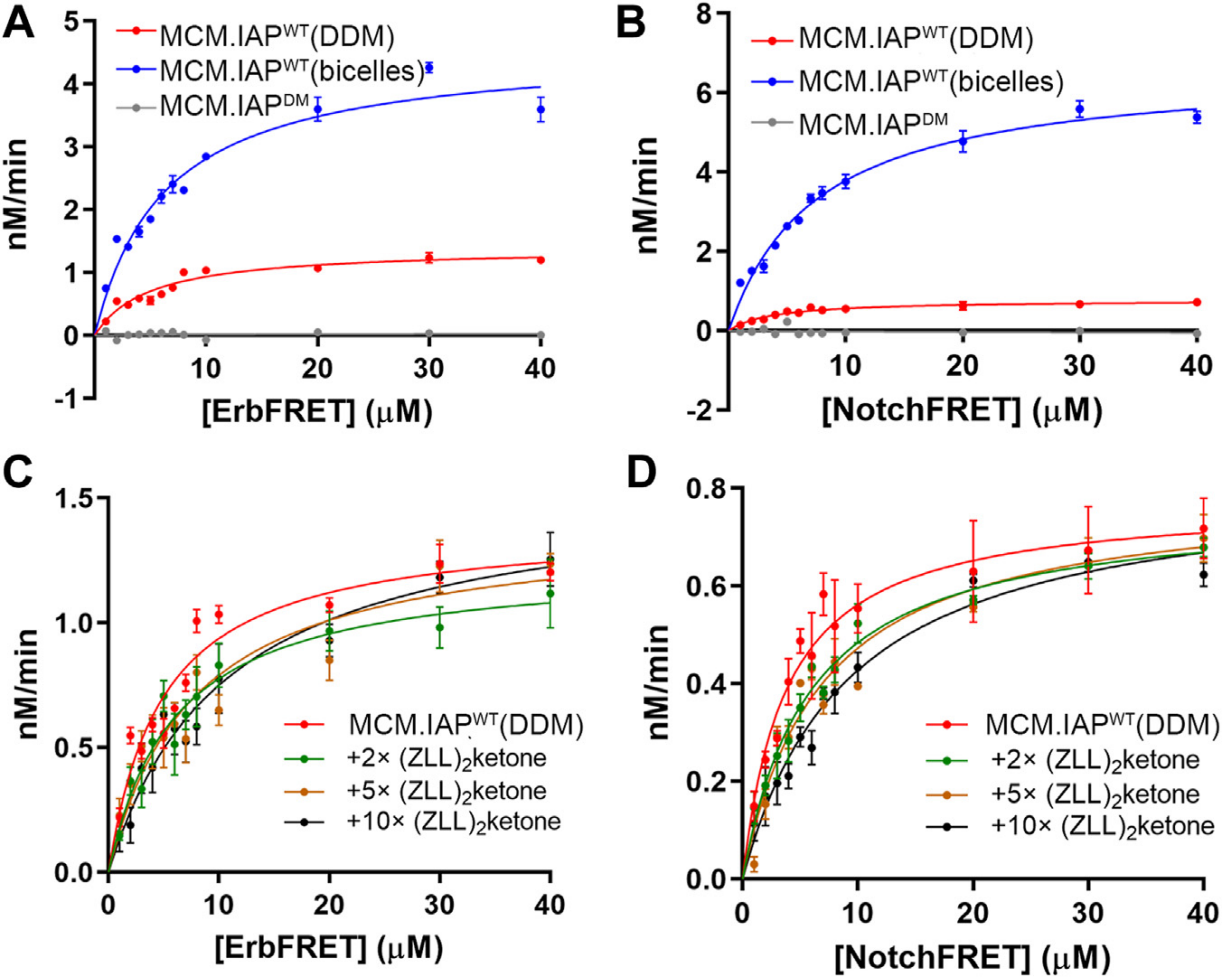
**FRET enzyme kinetics assays.***A*, Michaelis-Menten analysis of ErbFRET cleavage by MCM.IAP^WT^ in DDM and bicelle and by MCM.IAP^DM^ in DDM. *B*, Michaelis-Menten analysis of NotchFRET cleavage by MCM.IAP^WT^ in DDM and bicelles and by MCM.IAP^DM^ in DDM. *C*, kinetics data of ErbFRET cleavage by MCM.IAP^WT^ treated with increasing (ZLL)_2_ketone. *D*, kinetics data of NotchFRET cleavage by MCM.IAP^WT^ treated with increasing (ZLL)_2_ketone. See also Table 2 and Fig. S3.

MCM.IAP cleaves ErbFRET and NotchFRET in both DDM and bicelles (Fig. 4, *A* and *B*). MCM.IAP^DM^ is inactive against both substrates (Fig. 4, *A* and *B*), as anticipated. The compound 1,3-di-(N-carboxybenzoyl-L-leucyl-L-leucyl)amino acetone ((ZLL)_2_ketone) is a competitive inhibitor (Fig. 4, *C* and *D* and Table 2), seen as an increase in K_m_ but no change in V_max_. In sum, features of substrate access and binding stay the same across examined substrates here and previously (22, 24).

The kinetic parameters for ErbFRET and NotchFRET cleaved by MCM.IAP are similar to one another (Table 2) and in line with sluggish kinetics reported for other IAP substrates (22, 24) and other IPs like the serine IP rhomboid (32, 33). The apparent affinity, as proxied by K_m_, for ErbFRET and NotchFRET are similar to one another in DDM (4.9 ± 0.6 for ErbFRET *versus* 4.2 ± 0.5 μM for NotchFRET), and are weaker in bicelles (6.3 ± 0.6 μM for ErbFRET *versus* 8.3 ± 0.8 μM for NotchFRET, respectively). Both substrates exhibit a significant increase in V_max_ in bicelles as compared to DDM (V_max_ 2.8 ± 0.1 min^−1^ for ErbFRET and 2.0 ± 0.1 min^−1^ for NotchFRET in DDM *versus* 4.6 ± 0.2 min^−1^ for ErbFRET and 6.9 ± 0.3 min^−1^ for NotchFRET in bicelles). Accordingly, catalytic efficiencies for both substrates are improved in bicelles compared to DDM (0.57 ± 0.07 μM^−1^•min^−1^ for ErbFRET and 0.48 ± 0.06 μM^−1^•min^−1^ for NotchFRET in DDM *versus* 1.5 ± 0.20 μM^−1^•min^−1^ for ErbFRET and 1.7 ± 0.20 μM^−1^•min^−1^ for NotchFRET in bicelles), in line with the qualitative results from the gel-based assay showing accumulation of detectable product at earlier time points in bicelles compared to DDM (Figs. 2 and 3). This corresponds to a ∼3× improvement in catalytic efficiencies in bicelles (2.6-fold for ErbFRET and 3.5-fold for NotchFRET), the same as for MCM.IAP^WT^ acting on C100FRET (reported by us previously (24) and replicated independently here (Fig. S3), 3.0 ± 0.10 μM^−1^•min^−1^). However, the effect of bicelles on C100FRET cleavage by MCM.IAP is to increase the apparent affinity, namely, a more favorable K_m_ and only slight change in V_max_ (Table 2). Taken together, the lipid mimic affects the kinetic properties of MCM.IAP, in a substrate-dependent manner.

## Discussion

Intramembrane proteolysis must be highly regulated to prevent the cleavage of TM helices of off-target proteins and deactivation of the membrane-bound proteome of cells (34). Key studies, conducted over the past 30 years primarily in cell culture animal models, emphasize the incredible complexity of IAP cleavage: different IAP homologs can cleave non-biological substrates (17, 22) and there is no sequence recognition motif among the nearly 150 substrates identified for presenilin-1, just one of many human IAPs (6). Moreover, within a single substrate, APP, and with one enzyme, presenilin-1, multiple cut sites are thought to affect downstream Alzheimer's disease progression (35). Few studies intentionally reduce the complexity of the system to the fewest variables, as we did in the current study. By using a purified microbial ortholog, a focused set of substrates, and systematically changing lipid environments we show how both cleavage profiles and kinetic parameters are exquisitely affected by the lipid environment (Fig. 1). These studies lay the foundation for better comprehension of the chemical aspects of substrates that drive IAP processivity and kinetics in different contexts. Such inquiry lessens the knowledge gap of how IAP activity is regulated in the cell to control protein maturation and release of specific bioactive peptides at the right place and right time (34).

MCM.IAP cleaved MBP-HCV-ySUMO and MBP-ErbB4-ySUMO relatively selectively, at a single major cut site at the polar residue (Thr/Ser) present at position 6 or 7, with only minor adjacent cleavage products observed in either direction of the scissile bond. To our knowledge, no cleavage sites have been elucidated for ErbB4 and there is no consensus for cleavage sites for HCV. For the HCV, L-A and L-L were proposed in one of the earliest studies of SPP involvement in HCV core protein maturation, using the low-resolution mass spectrometry methods available at the time (20) whereas in a second study S-C was implicated in a previous study using site-direct mutagenesis, along with A-L (10), which we do not observe to an appreciable extent. Across the four different fusion protein substrates examined here, as well as those derived from Aβ examined in our earlier studies (Tables 1 and S2) (24), the most common cleavage positions were at positions 6 and 7. The observation that no major cleavage products are observed past position 7 in the four initial substrates is notable because cleavage at positions 9 to 10 was observed for the fortuitous substrate renin (Tables 1 and S2) (24) and in mutagenesis conducted with the Notch-1 sequence, so in principle, these residues are accessible to the enzyme. In sum, our expanded cleavage profiling of substrates indicates that cleavage is predominantly centered around Ser or Thr when present at position 6 or 7 as in HCV, ErbB4, and our Aβ substrates (Table S2) (22, 24), whereas the presence of Cys does not have a similar effect. Since so few biological γ-secretase substrates have Thr, Ser in register with the observed Thr or Ser cut sites for Aβ (Table S2), such a direct chemical preference for Ser or Thr at the scissile bond is likely a driver only for a small minority of substrates.

By contrast, MBP-CSFV-ySUMO and MBP-Notch-1-ySUMO were cleaved at multiple sites. Both substrates were cleaved at positions that include aromatic residues, W or F, closer to the N-terminal end of the substrate compared to the more precise cleavages for the other substrates. MBP-CSFV-ySUMO is unique in that it was the only substrate tested that was not cleaved to a large extent at position 6 or 7 and instead was primarily cleaved at 3 to 4. Cleavage of Notch-1 by γ-secretase has not been investigated to the extent as cleavage of APP, but previous literature indicates a cut site for γ-secretase around G7-V8 within the Notch-1 TM helix (14, 36), the same site we see in our assay. G7-V8 is in register with cleavage sites for two of our analogous Aβ-based MBP-ySUMO fusion substrates (Tables 1 and S2) (24), but the distribution is considerably broader than seen for MBP-ErbB4-ySUMO, MBP-HepC-ySUMO, or Aβ-based substrates (24).

Processive cleavage to release tri- and tetrapeptides is well documented for γ-secretase cutting Aβ substrates (37) but not, to our knowledge, for other substrates. Processive cleavage of Notch-1 by MCM.IAP is suggested by our Notch-1 mutagenesis experiments. For the C6V mutant, when the reaction is conducted in bicelles, a second G-V cut site is created and appears to turn off the original G7-V8 cut site. In turn, cleavage is seen in two clusters, at positions n and n-4. We did not observe cleavage patterns suggestive of processivity for the four other substrates reported in this study, or in our previous studies (22, 24). It is possible that elements of molecular recognition involving regions of the substrate beyond the isolated sequence used in our studies are responsible for controlling this aspect of cleavage for these substrates whereas for Notch-1, the information is encoded in the 10-mer sequence. Such recognition remains an open question. For example, the basic stretch of residues at the termini of the majority of TM segments (Table 1), which were found to form a hybrid β-sheet with γ-secretase (38, 39), are not present in our substrates, so these residues may be more important in orientation of the substrate as a whole rather than assisting in anchoring the substrate for a specific cleavage at the active site.

Early studies of IAPs proposed that substrate specificity was dictated by the presence of polar residues like Thr and Ser at the scissile bond, which destabilize the TM helix and expose the bond for proteolysis (40, 41). Comparison of experimental cleavage positions and hydropathy plots for the ten total 10-mer substrates examined here and previously (Fig. S5) coalesce around a tendency toward cleavage at not a polar or helix breaking residue but at a local minimum for hydrophobicity. This trend is readily apparent for HCV and ErbB4 (Fig. S4), for CSFV, which was cleaved at the hydropathy minimum at A3-W4, as well as for Notch1 and its variants, which were cleaved to a substantial extent at the hydropathy minimum at G5-C6 and C6-G7. For the three Aβ-based substrates (22, 24), cleavage is seen at hydropathy local minima starting from position 3 to 10 or 4 to 10, depending on the specific substrate. The only outlier is renin, which is also the lone non-biological substrate. Perhaps the initial hypothesis for IAP cleavage preference can be fine-tuned by broadening the scope to relative hydrophobicity in the vicinity of the scissile bond, a postulate that can be tested in future studies.

In addition to a proclivity toward the hydrophobicity minimum across substrates, a remarkable result from our study is the effect of the lipid membrane environment on enzyme kinetics. Different lipid compositions lead to membrane mimetics that affect the structure and dynamics of embedded proteins (42, 43), as well as alter the membrane permeability of small molecules such as water. In the gel-based assay, most products accumulated at relatively faster rates in DMPC/CHAPSO bicelles compared to DDM detergent solutions. Quantified in the corresponding 10-mer peptide FRET assay, ErbFRET and NotchFRET substrates, representing precise and promiscuous cleavage, respectively, are both cleaved by MCM.IAP with a ∼3× higher catalytic efficiency in bicelles compared to DDM, achieved primarily by an increase in V_max_. This increase in catalytic efficiency is the same as for C100FRET, achieved primarily by decreasing K_m_ in bicelles. Only the kinetics of the fortuitous reninFRET substrate, tested in our previous study (22) and not a native substrate, are inert to the lipid environment, despite the fact that catalytic parameters and FRET pairs are the same as for ErbFRET and NotchFRET. Perhaps *bona fide* substrates are affected by the lipid environment, for example, by changing accessibility or approach of the scissile bond, a testable hypothesis for the future.

While the sensitivity of γ-secretase activity to local membrane lipid composition is known (44, 45, 46, 47), the extent to which this affects catalytic rate as a general feature of IAPs was not previously appreciated. This raises the question of what specific membrane properties endowed by bicelles-physical (curvature, thickness), mechanical (stiffness), and chemical (polarity, saturation), underlie the effects of catalysis. γ-secretase localizes to cholesterol-rich lipid raft microdomains in the eukaryotic cell (48, 49, 50) and its activity is highly sensitive to membrane composition, including cholesterol (44, 45, 46, 47). Perhaps CHAPSO, a bile acid with overall chemical similarity to cholesterol, modulates MCM.IAP proteolysis by colocalizing enzyme and substrate in CHAPSO-rich regions of the bicelles (51). In support of this notion, our prior characterization of DDM/CHAPSO/DMPC bicelles indicates that these components are at least partially miscible (30). Direct binding of CHAPSO to MCM.IAP to modulate activity is unlikely, however, given that sterols are not present in archaea (52). In addition, while the APP TM segment binds cholesterol in a stoichiometric ratio, the binding site is not present in our FRET peptide where rate enhancement is observed in bicelles, and Notch-1 only weakly binds cholesterol (53). Alternatively, perhaps the hydrophilic moieties of CHAPSO play a role. CHAPSO could facilitate water entry to the active site or otherwise favorably interact with the substrate (54).

Our finding that for MCM.IAP, the DMPC-CHAPSO bicelle environment results in generally fewer cleavage products than in DDM micelles, particularly evident for SPP substrates, which is different from a study of APP cleavage by MCM.IAP in small unilamellar vesicles (SUVs) composed of 1-palmitoyl-2-oleoyl-glycero-3-phosphocholine (POPC), where processivity was enhanced compared to MCM.IAP in DDM micelles (55). The substrates and methodologies used in each study differ considerably, but differential results support the conclusion that the membrane properties affect cleavage profiles, with differences in thickness, curvature, and dynamics as possible culprits. In terms of thickness, in a cellular context, membrane lipid remodeling to adjust the thickness of the membrane decreased γ-secretase activity, and both overall decreased Aβ production and reduced the Aβ42/Aβ40 ratio (56). However, in our biochemical setup, DMPC/CHAPSO bicelles (30, 57) (25.6–25.7 Å) are only ∼3 Å thinner than POPC (28.8 Å) (57), suggesting this may not be a major factor. On the other hand, curvature is a key difference among DDM micelles, POPC vesicles, and bicelles. Whereas DDM micelles and POPC vesicles are highly curved (58), bicelles lack curvature in the hydrophobic center of the disc or ribbon (30). In support of higher processivity in POPC vesicles and DDM micelles compared to bicelles, molecular dynamics simulations predict that membrane curvature distorts TM helices to increase processivity (59). Differential dynamics, perhaps due to the presence of unsaturation in the alkyl chain of POPC *versus* the saturated chain in DMPC in bicelles, may further modulate processivity.

Overall, our results reveal chemical specificity hidden within IAP substrates and emphasize the way lipids regulate cleavage by tuning substrate profiles, processivity, and kinetics. Our findings motivate future directions related to dissecting the physicochemical basis for these phenomena. For example, comparing families of related substrates, *e.g.*, Notch-1–4 or tyrosine receptor kinases, which do not have identical transmembrane helices yet are all γ-secretase substrates, might be enlightening. Alternatively, focusing on a single substrate, and systematically changing the lipid composition may reveal new clues into the properties that control cleavage. Such studies should also provide insight into how bulk water molecules (24) access the membrane-embedded active site, as well as the driving forces for a hydrophobic TM helix to depart the lipid environment for a chemically incompatible hydrophilic active site (60). In the long term, such a basic biophysical inquiry should lead to a better ability to selectively target certain substrates for therapeutic application.

## Experimental procedures

### Molecular biology

MCM.IAP was cloned into pET-22b(+) vector (Novagen) as described previously (22), with an N-terminal pelB leader sequence for periplasmic membrane insertion and a C-terminal hexahistidine tag for affinity purification. Catalytically inactive double mutant (MCM.IAP^DM^) D162A/D220A plasmid was generated by site-directed mutagenesis (Table S3). Plasmid fidelity was obtained by DNA sequencing (Eton).

Fusion substrates were designed as we did previously for amyloid-β precursor protein (APP) γ- and ε-cleavage sites (24). Namely, substrates consisted of a 10-mer peptide insert containing a putative cleavage site, flanked by maltose binding protein and yeast small ubiquitin-like modifier (Table 1, MBP-XXXXXXXXXX-ySUMO). Corresponding plasmids were purchased from GenScript. Mutations were introduced by site-directed mutagenesis (Table S3). Plasmid fidelity was obtained by DNA sequencing (Genscript or Eton).

### Expression and purification of MCM.IAP

MCM.IAP (wild-type or double mutant) was expressed (22) in *E. coli* Rosetta 2 (DE3) cells (Novagen) and purified (24) as described previously. After Ni-affinity and size-exclusion chromatography, purity was assessed by 12% SDS-PAGE analysis and visualized by standard Coomassie staining (Fig. S5). Fractions containing MCM.IAP were pooled and concentrated using Amicon Ultra 15 ml centrifugal filter units (Millipore Sigma) with a molecular weight cut-off (MWCO) of 50 KDa and exchanged into PBS buffer (10 mM Na_2_HPO_4_/NaH_2_PO_4_, 150 mM NaCl, pH 7.2) supplemented with 0.05% n-dodecyl-β-D-maltoside (DDM). The concentration of MCM.IAP was calculated from Beer’s law using the absorption at 280 nm, the molecular weight, and the extinction coefficient calculated from ExPASy ProtParam (61). Freshly purified MCM.IAP was used immediately for downstream assays.

### Substrate expression and purification

Each substrate was expressed in *E. coli BL21 (DE3)* cells (Novagen) and purified in the absence of DDM as described previously (24). After Ni-affinity, MBPTrap affinity, and size-exclusion chromatography, purity was assessed by gel electrophoresis (Fig. S5) as described for MCM.IAP. Fractions containing pure protein were pooled and concentrated using Amicon Ultra 15 ml centrifugal filter units (Millipore Sigma) with a MWCO of 30K and exchanged into PBS buffer lacking DDM. The concentration of each substrate was calculated from the UV absorption at 280 nm, as described for MCM.IAP. The substrate was diluted to a stock concentration of 50 μM, aliquoted, and flash frozen by liquid nitrogen and stored at −80 °C until later use.

### Gel assay

Freshly purified MCM.IAP (16 μM) was incubated with each substrate (5 μM) separately at 37 °C over the course of 48 h. For experiments in bicelles, after buffer exchange, the purified protein was reconstituted into 5% (w/w) bicelle prepared from DMPC and CHAPSO, as described previously (28). At each time point, 5 μl of the reaction mixture was taken and quenched by an equal volume of 2× Laemmli sample buffer. At the conclusion of the experiments, each time point sample was run on a 12% SDS-PAGE gel with PageRuler Plus Prestained Protein Ladder (Thermo Scientific) and transferred onto a polyvinylidene difluoride (PVDF) membrane (Millipore). Standard Western blot procedures were carried out with 1:1000 MBP-probe mouse monoclonal IgG (Santa Cruz) as the primary antibody and 1:5000 HRP-conjugated goat anti-mouse monoclonal IgG (KPL) as the secondary antibody. The membrane was incubated with WesternBright ECL Spray (Advansta) and visualized by Amersham Imager 600 (GE Healthcare) or ChemiDoc Imaging System (Bio-Rad). At least three biological replicates for each substrate and condition were analyzed.

### Analysis of cleavage products by liquid chromatography-tandem mass spectrometry (LC-MS/MS)

For samples submitted for LC-MS/MS analysis, the gel assay was stopped at 48 h by the addition of 6.5× volume of acetone pre-chilled at −20 °C. The samples were incubated at −20 °C for 2 h. Acetone was removed, and the precipitated samples were resuspended in Laemmli sample buffer and analyzed by 12% SDS-PAGE. For gel assays conducted in bicelles, each precipitated sample was run separately on 2 to 3 lanes on an SDS-PAGE gel and the gel slices were pooled for additional processing. Gel slices containing cleavage band (∼40 kDa) were excised, pooled as appropriate, placed into a microcentrifuge tube, and dehydrated in 1 ml acetonitrile for 5 min. Acetonitrile was discarded and any remaining liquid evaporated in a SpeedVac for 10 min. To each tube, 400 μl of 100 mM ammonium bicarbonate and 4 μl of 500 mM DTT were then added and incubated for 30 min at room temperature. The solution was decanted and alkylation was initiated by the addition of 400 μl of 50 mM iodoacetamide for 30 min at room temperature. Iodoacetamide was decanted and the sample was washed with 1 ml of 50 mM ammonium bicarbonate for 10 min. After this wash was removed, 1 ml acetonitrile was added for 5 min, discarded, and then the sample was rehydrated by 400 μl of 100 mM ammonium bicarbonate for 10 min. Ammonium bicarbonate was removed and the gel slice was dehydrated by the addition of 800 μl and then 400 μl acetonitrile. The acetonitrile was removed and the material dried in a SpeedVac for 10 min.

In-gel digestion and LC-MS/MS were performed by the System Mass Spectrometry Core (Sym-C) at Georgia Institute of Technology or IDeA National Resource for Quantitative Proteomics at the University of Arkansas Medical School (AUMS) as follows. Glu-C (Promega) was prepared as a stock solution at 30 ng/μl in 100 mM ammonium bicarbonate. 100 μl Glu-C solution was added to each sample which was incubated overnight at 37 °C. The supernatant was saved in a microcentrifuge tube. The gel was extracted with 200 μl of 100 mM ammonium bicarbonate, vortexed, incubated for 10 min, centrifuged and the supernatant was kept in the previous tube. The peptides were extracted by 100 μl extraction solution (5% formic acid in 50% acetonitrile), incubated for 10 min, and the extract was kept in the same tube. The extraction was repeated and the final sample was evaporated to 20 μl.

At Sym-C, samples were analyzed by UltiMate 3000 RSLCnano UPLC system (Thermo) coupled to Q Exactive Plus Mass Spectrometer (Thermo Scientific). The Mascot Search engine (version 2.6.0) was used with Proteome Discoverer version 2.1 (Thermo Scientific). Peptide spectral matches with an expectation value < 0.01 (“High Confidence”) were used. At the IDeA National Resource for Quantitative Proteomics facility samples were analyzed by UltiMate 3000 RSLCnano UPLC system (Thermo) coupled to Orbitrap Eclipse Tribrid mass spectrometer (Thermo), and proteins were identified by database search using MaxQuant (Max Planck Institute). At least two biological replicates were analyzed for each substrate and condition. PSM values for the reporter peptide were converted to %PSM, and the 10-mer insert sequence was plotted in Figures 2 and 3.

### FRET enzyme kinetics assay

Custom FRET substrates containing 4-((4-(dimethylamino)phenyl)azo)benzoyl (Dabcyl) and N-[2-[(5-sulfo-1-naphthalenyl)amino]ethyl]-L-α-glutamine (EDANS) FRET pairs (Table 2) were custom synthesized by Biosynth. Corresponding FRET peptides for HepC and CSFV were too hydrophobic for synthesis (not shown). The lyophilized substrate was dissolved in DMSO to a stock concentration of 500 μM. For each substrate, the stock solution was sonicated by a water bath sonicator (Branson Ultrasonics Corp) covered by aluminum foil to maintain protection from light at 50 to 60 Hz, 117 V, 0.7 amp for 1 h. The solution was then covered by aluminum foil and incubated at 37 °C for 1 h to resolve high background. The stock was diluted to 1 to 40 μM solutions in PBS with 0.05% (w/v) DDM, and transferred into black-bottomed, non-binding 96-well plates (Corning Inc). The plates were covered by optical adhesive films (Micro-Amp) and read every 1 min at 37 °C in a Synergy 2 plate reader (BioTek, filters λex = 360 ± 40 nm, λem = 485 ± 20 nm), for at least 1 h until the fluorescence readings approached a plateau. Freshly purified MCM.IAP in PBS with 0.05% (w/v) DDM or reconstituted into 5% bicelle was then added, to achieve a final MCM.IAP concentration of 500 nM in each well. For blanks, PBS with 0.05% DDM or 5% empty bicelle was added without enzyme. For inhibition assays, inhibitor (ZLL)_2_ketone (EMD Millipore) was diluted in DMSO to a stock concentration of 1 mM. Freshly purified MCM.IAP in PBS with 0.05% (w/v) DDM was preincubated with (ZLL)_2_ketone at a 2 to 10× molar ratio to MCM.IAP at 37 °C for 1 h before running the kinetics assay. Assays with C100FRET (Calbiochem), which contains the 8-mer peptide GGVVIATV flanked by N-methyl anthranilate (Nma) fluorophore and lysine-modified with 2-nitrophenol (DNP) quencher, were conducted as previously reported (24). All kinetic data were analyzed using GraphPad Prism, plotting the initial slope as a function of substrate, as previously described (22). Data and statistics analysis presented include at least three biological replicates for determination of kinetic parameters and two biological replicates for inhibitor studies for each substrate and condition.

## Data availability

Any raw data used in this study not already included in Supporting Information will be shared upon reasonable request.

## Supporting information

This article contains supporting information (61, 62).

## Conflict of interest

The authors declare that they have no conflicts of interest with the contents of this article.

## References

[1] Hedstrom L. (2002). Serine protease mechanism and specificity. Chem. Rev..

[2] Kuhnle N., Dederer V., Lemberg M.K. (2019). Intramembrane proteolysis at a glance: from signalling to protein degradation. J. Cell Sci..

[3] Verhelst S.H.L. (2017). Intramembrane proteases as drug targets. FEBS J..

[4] Erez E., Fass D., Bibi E. (2009). How intramembrane proteases bury hydrolytic reactions in the membrane. Nature.

[5] Dunn B.M. (2002). Structure and mechanism of the pepsin-like family of aspartic peptidases. Chem. Rev..

[6] Guner G., Lichtenthaler S.F. (2020). The substrate repertoire of gamma-secretase/presenilin. Semin. Cell Dev. Biol..

[7] Dalbey R.E., Lively M.O., Bron S., Dijl J.M.V. (1997). The chemistry and enzymology of the type I signal peptidases. Protein Sci..

[8] Lemberg M.K., Bland F.A., Weihofen A., Braud V.M., Martoglio B. (2001). Intramembrane proteolysis of signal peptides: an essential step in the generation of HLA-E epitopes. J. Immunol..

[9] Fluhrer R., Grammer G., Israel L., Condron M.M., Haffner C., Friedmann E. (2006). A gamma-secretase-like intramembrane cleavage of TNFalpha by the GxGD aspartyl protease SPPL2b. Nat. Cell Biol..

[10] McLauchlan J., Lemberg M.K., Hope G., Martoglio B. (2002). Intramembrane proteolysis promotes trafficking of hepatitis C virus core protein to lipid droplets. EMBO J..

[11] Xu X. (2009). Gamma-secretase catalyzes sequential cleavages of the AbetaPP transmembrane domain. J. Alzheimers Dis..

[12] O'Brien R.J., Wong P.C. (2011). Amyloid precursor protein processing and Alzheimer's disease. Annu. Rev. Neurosci..

[13] Okochi M., Steiner H., Fukumori A., Tanii H., Tomita T., Tanaka T. (2002). Presenilins mediate a dual intramembranous gamma-secretase cleavage of Notch-1. EMBO J..

[14] Gu Y., Misonou H., Sato T., Dohmae N., Takio K., Ihara Y. (2001). Distinct intramembrane cleavage of the beta-amyloid precursor protein family resembling gamma-secretase-like cleavage of Notch. J. Biol. Chem..

[15] Sachan N., Sharma V., Mutsuddi M., Mukherjee A. (2023). Notch signalling: multifaceted role in development and disease. FEBS J..

[16] Li X., Dang S., Yan C., Gong X., Wang J., Shi Y. (2013). Structure of a presenilin family intramembrane aspartate protease. Nature.

[17] Torres-Arancivia C., Ross C.M., Chavez J., Assur Z., Dolios G., Mancia F. (2010). Identification of an archaeal presenilin-like intramembrane protease. PLoS One.

[18] Heimann M., Roman-Sosa G., Martoglio B., Thiel H.J., Rumenapf T. (2006). Core protein of pestiviruses is processed at the C terminus by signal peptide peptidase. J. Virol..

[19] Ni C.Y., Murphy M.P., Golde T.E., Carpenter G. (2001). gamma-Secretase cleavage and nuclear localization of ErbB-4 receptor tyrosine kinase. Science.

[20] Hussy P., Langen H., Mous J., Jacobsen H. (1996). Hepatitis C virus core protein: carboxy-terminal boundaries of two processed species suggest cleavage by a signal peptide peptidase. Virology.

[21] Merilahti J.A.M., Elenius K. (2019). Gamma-secretase-dependent signaling of receptor tyrosine kinases. Oncogene.

[22] Naing S.H., Vukoti K.M., Drury J.E., Johnson J.L., Kalyoncu S., Hill S.E. (2015). Catalytic properties of intramembrane aspartyl protease substrate hydrolysis evaluated using a FRET peptide cleavage assay. ACS Chem. Biol..

[23] UniProt C. (2023). UniProt: the universal protein knowledgebase in 2023. Nucleic Acids Res..

[24] Naing S.H., Kalyoncu S., Smalley D.M., Kim H., Tao X., George J.B. (2018). Both positional and chemical variables control *in vitro* proteolytic cleavage of a presenilin ortholog. J. Biol. Chem..

[25] Mossessova E., Lima C.D. (2000). Ulp1-SUMO crystal structure and genetic analysis reveal conserved interactions and a regulatory element essential for cell growth in yeast. Mol. Cell.

[26] Drapeau G.R., Boily Y., Houmard J. (1972). Purification and properties of an extracellular protease of Staphylococcus aureus. J. Biol. Chem..

[27] Breddam K., Meldal M. (1992). Substrate preferences of glutamic-acid-specific endopeptidases assessed by synthetic peptide substrates based on intramolecular fluorescence quenching. Eur. J. Biochem..

[28] Ujwal R., Bowie J.U. (2011). Crystallizing membrane proteins using lipidic bicelles. Methods.

[29] Li M., Morales H.H., Katsaras J., Kučerka N., Yang Y., Macdonald P.M. (2013). Morphological characterization of DMPC/CHAPSO bicellar mixtures: a combined SANS and NMR study. Langmuir.

[30] Leite W.C., Wu Y., Pingali S.V., Lieberman R.L., Urban V.S. (2022). Change in morphology of dimyristoylphosphatidylcholine/bile salt derivative bicelle assemblies with dodecylmaltoside in the disk and ribbon phases. J. Phys. Chem. Lett..

[31] Senes A., Gerstein M., Engelman D.M. (2000). Statistical analysis of amino acid patterns in transmembrane helices: the GxxxG motif occurs frequently and in association with beta-branched residues at neighboring positions. J. Mol. Biol..

[32] Arutyunova E., Panwar P., Skiba P.M., Gale N., Mak M.W., Lemieux M.J. (2014). Allosteric regulation of rhomboid intramembrane proteolysis. EMBO J..

[33] Arutyunova E., Jiang Z., Yang J., Kulepa A.N., Young H.S., Verhelst S. (2018). An internally quenched peptide as a new model substrate for rhomboid intramembrane proteases. Biol. Chem..

[34] Brown M., Ye J., Rawson R., Goldstein J. (2000). Regulated intramembrane proteolysis: a control mechanism conserved from bacteria to humans. Cell.

[35] Wolfe M.S. (2020). Unraveling the complexity of gamma-secretase. Semin. Cell Dev. Biol..

[36] Chandu D., Huppert S.S., Kopan R. (2006). Analysis of transmembrane domain mutants is consistent with sequential cleavage of Notch by gamma-secretase. J. Neurochem..

[37] Takami M., Nagashima Y., Sano Y., Ishihara S., Morishima-Kawashima M., Funamoto S. (2009). gamma-Secretase: successive tripeptide and tetrapeptide release from the transmembrane domain of beta-carboxyl terminal fragment. J. Neurosci..

[38] Yang G., Zhou R., Zhou Q., Guo X., Yan C., Ke M. (2019). Structural basis of Notch recognition by human gamma-secretase. Nature.

[39] Zhou R., Yang G., Guo X., Zhou Q., Lei J., Shi Y. (2019). Recognition of the amyloid precursor protein by human gamma-secretase. Science.

[40] Lemberg M.K., Martoglio B. (2002). Requirements for signal peptide peptidase-catalyzed intramembrane proteolysis. Mol. Cell.

[41] Kroos L., Akiyama Y. (2013). Biochemical and structural insights into intramembrane metalloprotease mechanisms. Biochim. Biophys. Acta.

[42] Dong H., Sharma M., Zhou H.-X., Cross T.A. (2012). Glycines: role in α-helical membrane protein structures and a potential indicator of native conformation. Biochemistry.

[43] Cross T.A., Sharma M., Yi M., Zhou H.-X. (2011). Influence of solubilizing environments on membrane protein structures. Trends Biochem. Sci..

[44] Fraering P.C., Ye W., Strub J.M., Dolios G., LaVoie M.J., Ostaszewski B.L. (2004). Purification and characterization of the human gamma-secretase complex. Biochemistry.

[45] Wrigley J.D., Schurov I., Nunn E.J., Martin A.C., Clarke E.E., Ellis S. (2005). Functional overexpression of gamma-secretase reveals protease-independent trafficking functions and a critical role of lipids for protease activity. J. Biol. Chem..

[46] Osenkowski P., Ye W., Wang R., Wolfe M.S., Selkoe D.J. (2008). Direct and potent regulation of gamma-secretase by its lipid microenvironment. J. Biol. Chem..

[47] Wahrle S., Das P., Nyborg A.C., McLendon C., Shoji M., Kawarabayashi T. (2002). Cholesterol-dependent gamma-secretase activity in buoyant cholesterol-rich membrane microdomains. Neurobiol. Dis..

[48] Simons K., Ikonen E. (1997). Functional rafts in cell membranes. Nature.

[49] Simons K., Ehehalt R. (2002). Cholesterol, lipid rafts, and disease. J. Clin. Invest..

[50] Silvius J.R. (2003). Role of cholesterol in lipid raft formation: lessons from lipid model systems. Biochim. Biophys. Acta.

[51] Zhdanov V.P., Höök F. (2015). Kinetics of enzymatic reactions in lipid membranes containing domains. Phys. Biol..

[52] Salvador-Castell M., Tourte M., Oger P.M. (2019). In search for the membrane regulators of archaea. Int. J. Mol. Sci..

[53] Deatherage C.L., Lu Z., Kroncke B.M., Ma S., Smith J.A., Voehler M.W. (2017). Structural and biochemical differences between the Notch and the amyloid precursor protein transmembrane domains. Sci. Adv..

[54] Mathai J.C., Tristram-Nagle S., Nagle J.F., Zeidel M.L. (2008). Structural determinants of water permeability through the lipid membrane. J. Gen. Physiol..

[55] Feilen L.P., Chen S.Y., Fukumori A., Feederle R., Zacharias M., Steiner H. (2022). Active site geometry stabilization of a presenilin homolog by the lipid bilayer promotes intramembrane proteolysis. Elife.

[56] Winkler E., Kamp F., Scheuring J., Ebke A., Fukumori A., Steiner H. (2012). Generation of Alzheimer disease-associated amyloid β42/43 peptide by γ-secretase can be inhibited directly by modulation of membrane thickness. J. Biol. Chem..

[57] Kucerka N., Nieh M.P., Katsaras J. (2011). Fluid phase lipid areas and bilayer thicknesses of commonly used phosphatidylcholines as a function of temperature. Biochim. Biophys. Acta.

[58] Hitzenberger M., Götz A., Menig S., Brunschweiger B., Zacharias M., Scharnagl C. (2020). The dynamics of γ-secretase and its substrates. Semin. Cell Dev. Biol..

[59] Dominguez L., Foster L., Straub J.E., Thirumalai D. (2016). Impact of membrane lipid composition on the structure and stability of the transmembrane domain of amyloid precursor protein. Proc. Natl. Acad. Sci. U. S. A..

[60] Liu X., Zhao J., Zhang Y., Ubarretxena-Belandia I., Forth S., Lieberman R.L. (2020). Substrate-enzyme interactions in intramembrane proteolysis: gamma-secretase as the prototype. Front. Mol. Neurosci..

[61] Gasteiger E., Hoogland C., Gattiker A., Duvaud S.E., Wilkins M.R., Appel R.D. (2005). Protein Identification and Analysis Tools on the ExPASy Server.

[62] Kyte J., Doolittle R.F. (1982). A simple method for displaying the hydropathic character of a protein. J. Mol. Biol..

