# Supplementary material for: Lipid environment modulates processivity and kinetics of a presenilin homolog acting on multiple substrates in vitro
Source: J Biol Chem. 2023 Oct 29;299(12):105401. doi: 10.1016/j.jbc.2023.105401 (PMC10679502; doi:10.1016/j.jbc.2023.105401)
Supplement: Supplemental Figures and Tables [file mmc1.pdf]

Supporting Information for

Lipid environment modulates processivity and kinetics of a presenilin homolog acting on multiple substrates in vitro

Yuqi Wu, Gwendell M. Thomas, Max Thomsen, Sara Bahri, Raquel L. Lieberman

School of Chemistry & Biochemistry, Georgia Institute of Technology, 901 Atlantic Dr. NW, Atlanta, GA 30332-0400

Table S1. LC/MS-MS data.

Table S2. Alignment of 10-mer peptides used in this and prior studies using MBP-XXXXXXXXXX-ySUMO.

Table S3. Primers for site-directed mutagenesis of MCM.IAPDM and MBP-Notch1-ySUMO substrate.

Figure S1. Sequence alignment of γ-secretase substrates using Clustal Omega. Less than 20 unique sequences can be aligned with the γ- (Aβ42) and ε- cut sites of APP.

Figure S2. Uncropped Western blots. (A) Full blot of Figure 1A. (B) Full blot of Figure 1B. (C) Full blot of Figure 1D. (D) Full blot of Figure 1E. (E) Full blot of Figure 1G. (F) Full blot of Figure 1H. (G) Full blot of Figure 1J. (H) Full blot of Figure 1H. (I) Full blot of Figure 2A. (J) Full blot of Figure 2B. (K) Full blot of Figure 2D. (L) Full blot of Figure 2E.

Figure S3. MCM.IAP^{WT} kinetic assays using C100FRET substrate. See Table 2 for kinetic parameters.

Figure S4. Normalized hyrophobicity plot using Kyte-Doolittle algorithm[1] within Expasy ProtScale[2], using a window of 3 residues.

Figure S5. SDS-PAGE purity assessments. (A) Purification of MCM.IAP. 1, MW marker. 2, MCM.IAPWT after nickel-affinity chromatography. 3, MCM.IAPWT after size-exclusion chromatography. 4, MCM.IAPDM after nickel-affinity chromatography. 5, MCM.IAPDM after size-exclusion chromatography. 6, MW marker. MCM.IAPWT and MCM.IAPDM have a MW of 31 kDa. (B) Purification of MBP fusion substrates, MBP-Notch-ySUMO as an example. 1, MBP-Notch-ySUMO after nickel-affinity chromatography. 2, MW marker. 3-5, MBP-Notch-ySUMO after MBPTrap affinity chromatography. MBP fusion substrates have a MW of 54 kDa.

Supporting References

Table S1. LC/MS-MS data. Red font denotes insert sequence, black font derives from the MBP-SUMO fusion setup.

No.	HepC Sequence	Length from L(LA)	Length from ALK	HepC DDM			
				Expt 1A	Expt 1B	Expt 2A	Expt 2B
1	ALKDAQTNSL	1	10	0	0	0	0
2	ALKDAQTNSLL	2	11	0	0	0	0
3	ALKDAQTNSLLA	3	12	1	0	0	0
4	ALKDAQTNSLLAL	4	13	0	1	0	0
5	ALKDAQTNSLLALL	5	14	3	3	2	3
6	ALKDAQTNSLLALLS	6	15	9	6	7	4
7	ALKDAQTNSLLALLSC	7	16	3	2	2	1
8	ALKDAQTNSLLALLSCL	8	17	0	0	0	0
9	ALKDAQTNSLLALLSCLT	9	18	0	0	0	0
10	ALKDAQTNSLLALLSCLTV	10	19	0	0	0	0
11	ALKDAQTNSLLALLSCLTVM		20	0	0	0	0
12	ALKDAQTNSLLALLSCLTVMS		21	0	0	0	0
13	ALKDAQTNSLLALLSCLTVMSD		22	0	0	0	0
14	ALKDAQTNSLLALLSCLTVMSDS		23	0	0	0	0

Control Peptides Sequence	Expt 1A	Expt 1B	Expt 2A	Expt 2B
AVNKDKPLGAVALKSYEEE	50	45	44	38
KDTGIKVTVEHPDKLEE	34	27	31	26
NYLLTDEGLE	10	10	8	7
LAKDPRIAATME	10	12	10	7
LAKEFLE	4	3	4	2

Table S1 cont. LC/MS-MS data. Red font denotes insert sequence, black font derives from the MBP-SUMO fusion setup.

No.	HepC Sequence	Length from L(LA)	Length from ALK	HepC Bicelles Expt 1A	HepC Bicelles Expt 1B	HepC Bicelles Expt 2A	HepC Bicelles Expt 2B
1	ALKDAQTNSL	1	10	0	0	0	0
2	ALKDAQTNSLL	2	11	6	6	0	0
3	ALKDAQTNSLLA	3	12	2	2	0	0
4	ALKDAQTNSLLAL	4	13	4	2	0	1
5	ALKDAQTNSLLALL	5	14	6	8	2	3
6	ALKDAQTNSLLALLS	6	15	82	84	9	10
7	ALKDAQTNSLLALLSC	7	16	5	7	0	0
8	ALKDAQTNSLLALLSCL	8	17	1	0	0	0
9	ALKDAQTNSLLALLSCLT	9	18	0	0	0	0
10	ALKDAQTNSLLALLSCLTV	10	19	0	0	0	0
11	ALKDAQTNSLLALLSCLTVM		20	0	0	0	0
12	ALKDAQTNSLLALLSCLTVMS		21	0	0	0	0
13	ALKDAQTNSLLALLSCLTVMSD		22	0	0	0	0
14	ALKDAQTNSLLALLSCLTVMSDS		23	0	0	0	0

Control Peptides Sequence	Expt 1A	Expt 1B	Expt 2A	Expt 2B
AVNKDKPLGAVALKSYEEE	329	346	69	38
KDTGIKVTVEHPDKLEE	122	117	21	15
NYLLTDEGLE	22	24	12	5
LAKDPRIAATME	143	168	25	13
LAKEFLE	21	30	4	5

Table S1 cont. LC/MS-MS data. Red font denotes insert sequence, black font derives from the MBP-SUMO fusion setup.

No.	SwineFever Sequence	Length from L(LA)	Length from ALK	SwineFever DDM Expt 1A	SwineFever DDM Expt 1B	SwineFever DDM Expt 2A	SwineFever DDM Expt 2B
1	ALKDAQTNSL	1	10	0	0	2	2
2	ALKDAQTNSLL	2	11	0	0	7	8
3	ALKDAQTNSLLA	3	12	3	3	91	79
4	ALKDAQTNSLLAW	4	13	2	1	15	17
5	ALKDAQTNSLLAWA	5	14	5	3	96	106
6	ALKDAQTNSLLAWAV	6	15	1	1	2	2
7	ALKDAQTNSLLAWAVI	7	16	0	0	3	3
8	ALKDAQTNSLLAWAVIT	8	17	0	1	20	13
9	ALKDAQTNSLLAWAVITI	9	18	0	0	3	1
10	ALKDAQTNSLLAWAVITIL	10	19	0	0	1	1
11	ALKDAQTNSLLAWAVITILM		20	0	0	0	0
12	ALKDAQTNSLLAWAVITILMS		21	0	0	0	0
13	ALKDAQTNSLLAWAVITILMSD		22	0	0	0	0
14	ALKDAQTNSLLAWAVITILMSDS		23	0	0	0	0

Control Peptides Sequence	Expt 1A	Expt 1B	Expt 2A	Expt 2B
AVNKDKPLGAVALKSYEEE	59	61	431	445
KDTGIKVTVEHPDKLEE	37	41	182	185
NYLLTDEGLE	10	9	11	17
LAKDPRIAATME	12	15	104	109
LAKEFLE	5	4	5	5

Table S1 cont. LC/MS-MS data. Red font denotes insert sequence, black font derives from the MBP-SUMO fusion setup.

No.	SwineFever Sequence	Length from L(LA)	Length from ALK	SwineFever Bicelles			
				Expt 1A	Expt 1B	Expt 2A	Expt 2B
1	ALKDAQTNSL	1	10	0	2	0	0
2	ALKDAQTNSLL	2	11	5	6	6	8
3	ALKDAQTNSLLA	3	12	45	39	8	16
4	ALKDAQTNSLLAW	4	13	10	11	25	20
5	ALKDAQTNSLLAWA	5	14	5	4	14	8
6	ALKDAQTNSLLAWAV	6	15	10	14	0	0
7	ALKDAQTNSLLAWAVI	7	16	4	3	0	1
8	ALKDAQTNSLLAWAVIT	8	17	0	0	0	0
9	ALKDAQTNSLLAWAVITI	9	18	0	0	0	0
10	ALKDAQTNSLLAWAVITIL	10	19	0	0	0	0
11	ALKDAQTNSLLAWAVITILM		20	0	0	0	0
12	ALKDAQTNSLLAWAVITILMS		21	0	0	0	0
13	ALKDAQTNSLLAWAVITILMSD		22	0	0	0	0
14	ALKDAQTNSLLAWAVITILMSDS		23	0	0	0	0

Control Peptides Sequence	Expt 1A	Expt 1B	Expt 2A	Expt 2B
AVNKDKPLGAVALKSYEEE	340	418	427	419
KDTGIKVTVEHPDKLEE	136	135	130	130
NYLLTDEGLE	24	28	36	37
LAKDPRIAATME	164	202	231	241
LAKEFLE	24	34	40	35

Table S1 cont. LC/MS-MS data. Red font denotes insert sequence, black font derives from the MBP-SUMO fusion setup.

No.	ErbB4 Sequence	Length from L(VI)	Length from ALK	ErbB4 DDM			
				Expt 1A	Expt 1B	Expt 2A	Expt 2B
1	ALKDAQTNSL	1	10	2	1	1	0
2	ALKDAQTNSLV	2	11	0	0	1	0
3	ALKDAQTNSLVI	3	12	0	0	0	0
4	ALKDAQTNSLVIV	4	13	2	3	6	4
5	ALKDAQTNSLVIVG	5	14	4	4	6	5
6	ALKDAQTNSLVIVGL	6	15	45	23	8	9
7	ALKDAQTNSLVIVGLT	7	16	90	40	23	23
8	ALKDAQTNSLVIVGLTF	8	17	10	17	2	0
9	ALKDAQTNSLVIVGLTFA	9	18	3	3	1	0
10	ALKDAQTNSLVIVGLTFAV	10	19	1	0	0	0
11	ALKDAQTNSLVIVGLTFAVM		20	2	1	0	0
12	ALKDAQTNSLVIVGLTFAVMS		21	0	0	0	0
13	ALKDAQTNSLVIVGLTFAVMSD		22	0	0	0	0
14	ALKDAQTNSLVIVGLTFAVMSDS		23	0	0	0	0

Control Peptides Sequence	Expt 1A	Expt 1B	Expt 2A	Expt 2B
AVNKDKPLGAVALKSYEEE	518	282	120	69
KDTGIKVTVEHPDKLEE	189	76	26	24
NYLLTDEGLE	57	10	21	8
LAKDPRIAATME	173	46	24	28
LAKEFLE	23	25	6	5

Table S1 cont. LC/MS-MS data. Red font denotes insert sequence, black font derives from the MBP-SUMO fusion setup.

No.	ErbB4 Sequence	Length from L(VI)	Length from ALK	ErbB4 Bicelles Expt 1A	ErbB4 Bicelles Expt 1B	ErbB4 Bicelles Expt 2A	ErbB4 Bicelles Expt 2B
1	ALKDAQTNSL	1	10	0	0	3	5
2	ALKDAQTNSLV	2	11	0	0	0	0
3	ALKDAQTNSLVI	3	12	0	0	0	0
4	ALKDAQTNSLVIV	4	13	2	2	4	3
5	ALKDAQTNSLVIVG	5	14	2	2	10	10
6	ALKDAQTNSLVIVGL	6	15	10	11	178	170
7	ALKDAQTNSLVIVGLT	7	16	18	25	137	148
8	ALKDAQTNSLVIVGLTF	8	17	3	2	0	0
9	ALKDAQTNSLVIVGLTFA	9	18	2	0	0	0
10	ALKDAQTNSLVIVGLTFAV	10	19	0	0	0	0
11	ALKDAQTNSLVIVGLTFAVM		20	4	0	0	0
12	ALKDAQTNSLVIVGLTFAVMS		21	0	0	0	0
13	ALKDAQTNSLVIVGLTFAVMSD		22	0	0	0	0
14	ALKDAQTNSLVIVGLTFAVMSDS		23	0	0	0	0

Control Peptides Sequence	Expt 1A	Expt 1B	Expt 2A	Expt 2B
AVNKDKPLGAVALKSYEEE	123	62	575	592
KDTGIKVTVEHPDKLEE	27	20	324	267
NYLLTDEGLE	18	8	43	41
LAKDPRIAATME	29	25	373	379
LAKEFLE	4	5	55	56

Table S1 cont. LC/MS-MS data. Red font denotes insert sequence, black font derives from the MBP-SUMO fusion setup.

No.	Notch1 Sequence	Length from L(FF)	Length from ALK	Notch1 DDM Expt 1A	Notch1 DDM Expt 1B	Notch1 DDM Expt 2A	Notch1 DDM Expt 2B
1	ALKDAQTNSL	1	10	1	1	0	0
2	ALKDAQTNSLF	2	11	6	5	2	4
3	ALKDAQTNSLFF	3	12	2	0	0	0
4	ALKDAQTNSLFFV	4	13	0	0	0	0
5	ALKDAQTNSLFFVG	5	14	7	5	6	3
6	ALKDAQTNSLFFVGC	6	15	7	7	6	3
7	ALKDAQTNSLFFVGCG	7	16	9	9	7	4
8	ALKDAQTNSLFFVGCGV	8	17	0	0	0	0
9	ALKDAQTNSLFFVGCGVL	9	18	2	1	1	0
10	ALKDAQTNSLFFVGCGVLL	10	19	0	0	0	0
11	ALKDAQTNSLFFVGCGVLLM		20	0	0	0	0
12	ALKDAQTNSLFFVGCGVLLMS		21	0	0	0	0
13	ALKDAQTNSLFFVGCGVLLMSD		22	0	0	0	0
14	ALKDAQTNSLFFVGCGVLLMSDS		23	0	0	0	0

Control Peptides Sequence	Expt 1A	Expt 1B	Expt 2A	Expt 2B
AVNKDKPLGAVALKSYEEE	204	58	81	50
KDTGIKVTVEHPDKLEE	31	19	21	5
NYLLTDEGLE	21	7	7	3
LAKDPRIAATME	48	26	32	2
LAKEFLE	5	6	8	4

Table S1 cont. LC/MS-MS data. Red font denotes insert sequence, black font derives from the MBP-SUMO fusion setup.

No.	Notch1 Sequence	Length from L(FF)	Length from ALK	Notch1 Bicelles Expt 1A	Expt 1B	Expt 2A	Expt 2B
1	ALKDAQTNSL	1	10	2	1	5	4
2	ALKDAQTNSLF	2	11	5	5	50	53
3	ALKDAQTNSLFF	3	12	0	0	3	1
4	ALKDAQTNSLFFV	4	13	2	2	0	0
5	ALKDAQTNSLFFVG	5	14	7	6	8	7
6	ALKDAQTNSLFFVGC	6	15	5	4	16	18
7	ALKDAQTNSLFFVGCG	7	16	5	4	19	19
8	ALKDAQTNSLFFVGCGV	8	17	3	2	0	0
9	ALKDAQTNSLFFVGCGVL	9	18	2	0	1	3
10	ALKDAQTNSLFFVGCGVLL	10	19	0	0	0	0
11	ALKDAQTNSLFFVGCGVLLM		20	4	0	1	1
12	ALKDAQTNSLFFVGCGVLLMS		21	0	0	0	0
13	ALKDAQTNSLFFVGCGVLLMSD		22	0	0	0	0
14	ALKDAQTNSLFFVGCGVLLMSDS		23	0	0	0	0

Control Peptides Sequence	Expt 1A	Expt 1B	Expt 2A	Expt 2B
AVNKDKPLGAVALKSYEEE	97	54	529	586
KDTGIKVTVEHPDKLEE	15	12	267	254
NYLLTDEGLE	21	6	39	46
LAKDPRIAATME	32	29	328	347
LAKEFLE	5	6	41	50

Table S1 cont. LC/MS-MS data. Red font denotes insert sequence, black font derives from the MBP-SUMO fusion setup.

No.	Notch1_F2A Sequence	Length from L(AF)	Length from ALK	Notch1_F2A DDM Expt 1	Notch1_F2A DDM Expt 2	Notch1_F2A DDM Expt 3
1	ALKDAQTNSL	1	10	0	3	0
2	ALKDAQTNSLA	2	11	0	2	5
3	ALKDAQTNSLAF	3	12	0	0	0
4	ALKDAQTNSLAFV	4	13	0	2	3
5	ALKDAQTNSLAFVG	5	14	41	41	33
6	ALKDAQTNSLAFVGC	6	15	25	20	20
7	ALKDAQTNSLAFVGCG	7	16	24	19	19
8	ALKDAQTNSLAFVGCGV	8	17	21	1	9
9	ALKDAQTNSLAFVGCGVL	9	18	0	1	1
10	ALKDAQTNSLAFVGCGVLL	10	19	0	2	0
11	ALKDAQTNSLAFVGCGVLLM		20	0	0	0
12	ALKDAQTNSLAFVGCGVLLMS		21	0	0	0
13	ALKDAQTNSLAFVGCGVLLMSD		22	0	0	0
14	ALKDAQTNSLAFVGCGVLLMSDS		23	0	0	0

Control Peptides Sequence	Expt 1	Expt 2	Expt 3
AVNKDKPLGAVALKSYEEE	227	217	208
KDTGIKVTVEHPDKLEE	147	44	66
NYLLTDEGLE	18	19	14
LAKDPRIAATME	94	49	58
LAKEFLE	4	7	19

Table S1 cont. LC/MS-MS data. Red font denotes insert sequence, black font derives from the MBP-SUMO fusion setup.

No.	Notch1_F2A Sequence	Length from L(AF)	Length from ALK	Notch1_F2A Bicelles Expt 1	Notch1_F2A Bicelles Expt 2A	Notch1_F2A Bicelles Expt 2B
1	ALKDAQTNSL	1	10	0	0	0
2	ALKDAQTNSLA	2	11	0	0	0
3	ALKDAQTNSLAF	3	12	0	0	0
4	ALKDAQTNSLAFV	4	13	0	0	0
5	ALKDAQTNSLAFVG	5	14	40	13	13
6	ALKDAQTNSLAFVGC	6	15	25	9	8
7	ALKDAQTNSLAFVGCG	7	16	23	9	8
8	ALKDAQTNSLAFVGCGV	8	17	0	2	3
9	ALKDAQTNSLAFVGCGVL	9	18	0	0	0
10	ALKDAQTNSLAFVGCGVLL	10	19	0	0	0
11	ALKDAQTNSLAFVGCGVLLM		20	0	0	0
12	ALKDAQTNSLAFVGCGVLLMS		21	0	0	0
13	ALKDAQTNSLAFVGCGVLLMSD		22	0	0	0
14	ALKDAQTNSLAFVGCGVLLMSDS		23	0	0	0

Control Peptides Sequence	Expt 1	Expt 2A	Expt 2B
AVNKDKPLGAVALKSYEEE	453	176	200
KDTGIKVTVEHPDKLEE	184	33	30
NYLLTDEGLE	52	4	3
LAKDPRIAATME	155	36	38
LAKEFLE	11	7	6

Table S1 cont. LC/MS-MS data. Red font denotes insert sequence, black font derives from the MBP-SUMO fusion setup.

No.	Notch1_C6V Sequence	Length from L(FF)	Length from ALK	Notch1_C6V DDM				
				Expt 1A	Expt 1B	Expt 2	Expt 3	Expt 4
1	ALKDAQTNSL	1	10	0	1	0	0	0
2	ALKDAQTNSLF	2	11	7	6	0	5	13
3	ALKDAQTNSLFF	3	12	0	0	0	0	0
4	ALKDAQTNSLFFV	4	13	0	0	0	0	0
5	ALKDAQTNSLFFVG	5	14	16	13	4	3	7
6	ALKDAQTNSLFFVGV	6	15	9	9	4	12	10
7	ALKDAQTNSLFFVGVG	7	16	0	0	0	2	0
8	ALKDAQTNSLFFVGVGV	8	17	19	19	4	3	6
9	ALKDAQTNSLFFVGVGVL	9	18	1	0	0	1	1
10	ALKDAQTNSLFFVGVGVLL	10	19	0	0	0	0	1
11	ALKDAQTNSLFFVGVGVLLM		20	0	0	0	0	0
12	ALKDAQTNSLFFVGVGVLLMS		21	0	0	0	0	0
13	ALKDAQTNSLFFVGVGVLLMSD		22	0	0	0	0	0
14	ALKDAQTNSLFFVGVGVLLMSDS		23	0	0	0	0	0

Control Peptides Sequence	Expt 1A	Expt 1B	Expt 2	Expt 3	Expt 4
AVNKDKPLGAVALKSYEEE	374	356	44	38	84
KDTGIKVTVEHPDKLEE	213	198	31	26	47
NYLLTDEGLE	88	80	8	7	18
LAKDPRIAATME	45	40	10	7	45
LAKEFLE	0	0	4	2	5

Table S1 cont. LC/MS-MS data. Red font denotes insert sequence, black font derives from the MBP-SUMO fusion setup.

No.	Notch1_C6V Sequence	Length from L(FF)	Length from ALK	Notch1_C6V Bicelles Expt 1A	Expt 1B	Expt 2	Expt 3	Expt 4
1	ALKDAQTNSL	1	10	4	1	6	4	8
2	ALKDAQTNSLF	2	11	1	1	2	5	3
3	ALKDAQTNSLFF	3	12	2	0	6	0	0
4	ALKDAQTNSLFFV	4	13	0	0	0	0	2
5	ALKDAQTNSLFFVG	5	14	2	2	3	4	6
6	ALKDAQTNSLFFVGV	6	15	2	2	6	3	5
7	ALKDAQTNSLFFVGVG	7	16	0	0	6	0	0
8	ALKDAQTNSLFFVGVGV	8	17	0	0	0	0	0
9	ALKDAQTNSLFFVGVGVL	9	18	2	0	3	1	1
10	ALKDAQTNSLFFVGVGVLL	10	19	0	0	0	0	0
11	ALKDAQTNSLFFVGVGVLLM		20	0	0	0	0	0
12	ALKDAQTNSLFFVGVGVLLMS		21	0	0	0	0	0
13	ALKDAQTNSLFFVGVGVLLMSD		22	0	0	0	0	0
14	ALKDAQTNSLFFVGVGVLLMSDS		23	0	0	0	0	0

Control Peptides Sequence	B1	B1A2	B2	B3	B4
AVNKDKPLGAVALKSYEEE	84	63	38	45	68
KDTGIKVTVEHPDKLEE	47	22	20	22	71
NYLLTDEGLE	18	9	3	2	9
LAKDPRIAATME	45	34	28	30	63
LAKEFLE	5	5	0	2	2

Table S2. Alignment of 10-mer peptides used in this and prior studies using MBP-XXXXXXXXXX-ySUMO[a].

Substrate Name	10-mer sequence<br>12345678910
MBP-HCV-ySUMO (this study)	LLALLSCLTV
MBP-CSFV-ySUMO (this study)	LLAWAVITIL
MBP-ErbB4-ySUMO (this study)	LVIVGLTFAV
MBP-Notch1-ySUMO (this study)	LFFVGCGVLL
MBP-Notch1(F2A)-ySUMO (this study)	LAFVGCGVLL
MBP-Notch1(C6V)-ySUMO (this study)	LFFVGVGVLL
MBP-Aβγ-ySUMO (Naing 2018)	GGVVIATVIV
MBP-Aβ**ε**(v1)-ySUMO (Naing 2018)	TVIVITLVML
MBP-Aβ**ε**(v2)-ySUMO (Naing 2018)	GGVIVITLVM
MBP-renin-ySUMO (Naing 2015)	IHPFHLVIHT

[a]Sequence:

MKIEEGKLVIWINGDKGYNGLAEVGKKFEKDTGIKVTVEHPDKLEEKFPQVAATGDGPDIIFWA
HDRFGGYAQSGLLAEITPDKAFQDKLYPFTWDAVRYNGKLIAYPIAVEALSLIYNKDLLPNPPK
TWEEIPALDKELKAKGKSALMFNLQEPYFTWPLIAADGGYAFKYENGKYDIKDVGVDNAGAKAG
LTFLVDLIKNKHMNADTDYSIAEAAFNKGETAMTINGPWAWSNIDTSKVNYGVTVLPTFKGQPS
KPFVGVLSAGINAASPNKELAKEFLENYLLTDEGLEAVNKDKPLGAVALKSYEEELVKDPRIAA
TMENAQKGEIMPNIPQMSAFWYAVRTAVINAASGRQTVDE|ALKDAQTNSGGVVIATVIVMSDS
|EVNQEAKPEVKPEVKPETHINLKVSDGSSEIFFKIKKTTPLRRLMEAFAKRQGKEMDSLRFLY
DGIRIQADQTPEDLDMEDNDIIEAHREQIGGATYHHHHHH

Key: MBP, linker, substrate, ySUMO, | start/end of reporter peptide. See also Figure 1B.

Table S3. Primers for site-directed mutagenesis of MCM.IAPDM and MBP-Notch1-ySUMO substrate.

Primers for mutagenesis	Primer (5' to 3')
MCM.IAP D162A	Forward: CTCGCGGTCTACGCCGCCATATCGCTC
	Reverse: GACCGATATGGCGGCGTAGACCGCGAG
MCM.IAP D220A	Forward: GTCATGGGTATGGGCGCTCTCATCATGCC
	Reverse: GGCATGATGAGAGCGCCCATACCCATGAC
MBP-Notch1-F2A-ySUMO	Forward: CAGACTAATTCGCTGGCCTTTGTGGGCTGCGG
	Reverse: CCGCAGCCCACAAAGGCCAGCGAATTAGTCTG
MBP-Notch1-C6V-ySUMO	Forward: CTGTTTTTTGTGGGCGTGGGCGTGCTGCTGATG
	Reverse: CATCAGCAGCACGCCCACGCCCACAAAAAACAG

```
APP_Human__P05067_            1  -----GAIIGLM----VGGVVIATVIVI--TLVM----LKKK-----------   27
IL6R_Human__P08887_           1  -----TFLVAGG---SLAFG---TLLCI--AIVL----RFKKT----------   26
Jagged1_Rat__Q63722_          1  --FLVPLLSSVL----TVAW---VCCLV--TAFY----WCVRKRRRK------   32
IFNaR2_Human__P48551_         1  -------IGGII----TVFL---IALVL--TSTI----VTL------------   21
ErbB4_Human__Q15303_          1  -----LIAAGVI----GGLF---ILVIVGLTFAV----YVRRKSIKKKRALRR   37
CD200_Human__P41217_          1  YWFSVPLLLSIV----SLVI---LLVLI--SILL----YWYEKRRKP------   34
LRP1b_Human__Q9NZR2_          1  --------AIIV----PLVL---LVTLI--TTLVIGLVLCKRKRRTK------   30
APLP1_Mouse__Q03157_          1  ------ALSGLL----IMGAGGGSLIVL--SLLL----LRKKK----------   27
APLP2_Human__Q06481_          1  -----SALIGLL----VIAVAIATVIVI--SLVM----LRKRQ----------   28
APLP2_Mouse__Q06335_          1  -----NALIGLL----VIAVAIATVIVI--SLVM----LRKRQ----------   28
APLP1_Human__P51693_          1  ------AVSGLL----IMGAGGGSLIVL--SMLL----LRKKK----------   27
VE-cadherin_Human__P33151     1  ------AVVAIL----LCIL---TITVI--TLLI----FLRRRLRK-------   27
TRKA_Human__P04629_           1  --------LAVF----AC------LFLS--TLLL----VLNKCGRR-------   22
BCMA_Mouse__O88472_           1  ------VLWIFL----GLTL---VLSLALFTISF----LLRKM----------   26
MUSK_Human__O15146_           1  ------VIISIM----SSFA---IFVLLTITTLY----CCRRRK---------   27
Klotho_Human__Q9UEF7_         1  -------LLAFI----AFLF---FASIISLSLIF----YYSKKGRR-------   28
IL-1R1_Human__P14778_         1  ------HMIGIC----VTLT---VIIVC--SVFI----YKIFKI---------   25
Protogenin_Chicken__Q589G     1  -----GIVVGVC----IALT---CILIC-ILILI----YRSKAR---------   27
Ephrin-B1_Mouse__P52795_      1  ----VALFAAVGAGCVIFLL---IIIFL--TVLL----LKLRKRHRK------   34
Ephrin-B1_Human__P98172_      1  ----VALFAAVGAGCVIFLL---IIIFL--TVLL----LKLRKRHRK------   34
Robo1_Human__Q9Y6N7_          1  -----AFIAGIG---AACWI---ILMVF--SIWL----YRHRKKR--------   28
```

Figure S1. Sequence alignment of γ-secretase substrates using Clustal Omega. Less than 20 unique sequences can be aligned with the γ- ($A\beta_{42}$) and ε- cleavage sites of APP.

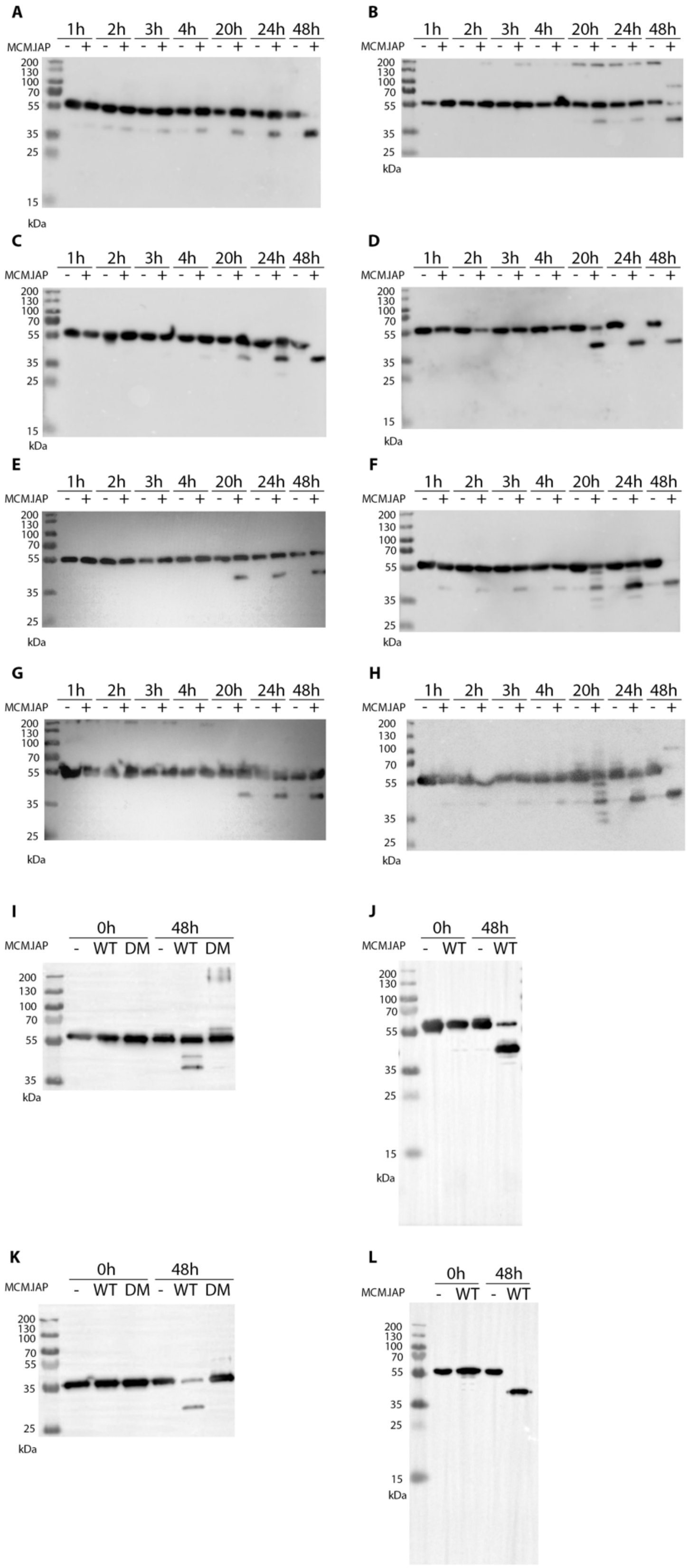

Figure S2. Uncropped Western blots. (A) Full blot of Figure 1A. (B) Full blot of Figure 1B. (C) Full blot of Figure 1D. (D) Full blot of Figure 1E. (E) Full blot of Figure 1G. (F) Full blot of Figure 1H. (G) Full blot of Figure 1J. (H) Full blot of Figure 1H. (I) Full blot of Figure 2A. (J) Full blot of Figure 2B. (K) Full blot of Figure 2D. (L) Full blot of Figure 2E.

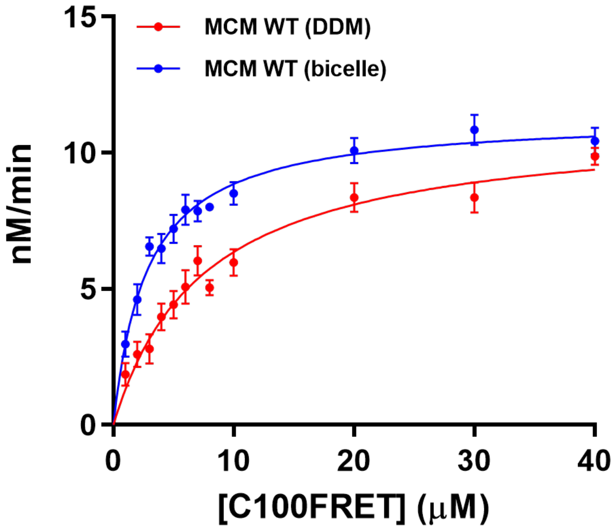

Figure S3 MCM.IAP^{WT} kinetic assays using C100FRET substrate. See Table 2 for kinetic parameters.

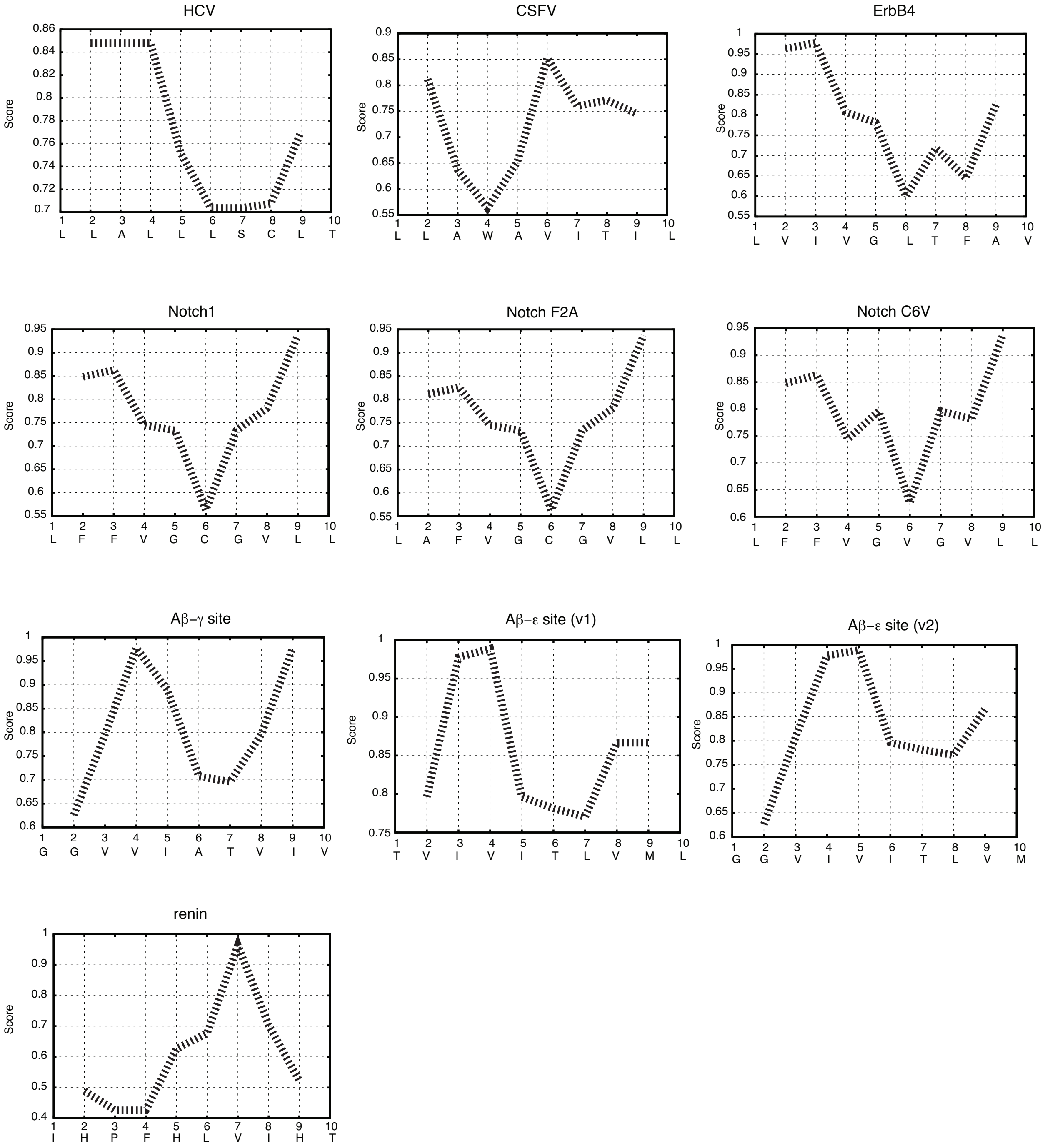

Figure S4. Normalized hyrophobicity plot using Kyte-Doolittle algorithm[1] within Expasy ProtScale[2], using a window of 3 residues.

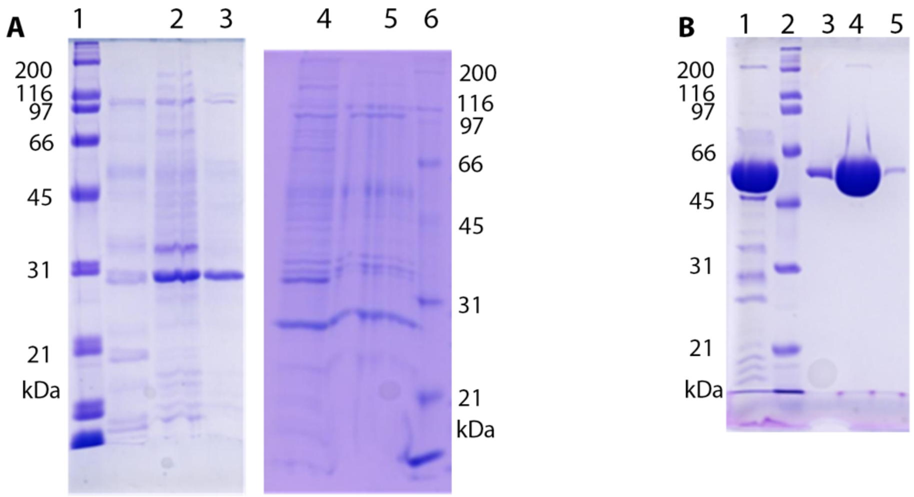

Figure S5. SDS-PAGE purity assessments. (A) Purification of MCM.IAP. 1, MW marker. 2, $MCM.IAP^{WT}$ after nickel-affinity chromatography. 3, $MCM.IAP^{WT}$ after size-exclusion chromatography. 4, $MCM.IAP^{DM}$ after nickel-affinity chromatography. 5, $MCM.IAP^{DM}$ after size-exclusion chromatography. 6, MW marker. $MCM.IAP^{WT}$ and $MCM.IAP^{DM}$ have a MW of 31 kDa. (B) Purification of MBP fusion substrates, MBP-Notch-ySUMO as an example. 1, MBP-Notch-ySUMO after nickel-affinity chromatography. 2, MW marker. 3-5, MBP-Notch-ySUMO after MBPTrap affinity chromatography. MBP fusion substrates have a MW of 54 kDa.

Supporting References

(1) Kyte, J.; Doolittle, R. F. A simple method for displaying the hydropathic character of a protein. *J Mol Biol* **1982**, *157* (1), 105-132.
(2) Gasteiger, E.; Hoogland, C.; Gattiker, A.; Duvaud, S. E.; Wilkins, M. R.; Appel, R. D.; Bairoch, A. Protein Identification and Analysis Tools on the ExPASy Server. Humana Press, 2005; pp 571-607.
